# Supplementary material for: A protocol for MIndfulness-based Neurofeedback to augment DBT psychotherapy for adults with Borderline Personality Disorder (MIND-BPD)
Source: PLoS One. 2026 Mar 20;21(3):e0338002. doi: 10.1371/journal.pone.0338002 (PMC13004318; doi:10.1371/journal.pone.0338002)
Supplement: S1 File — (PDF) [file pone.0338002.s001.pdf]

CLINICAL STUDY PROTOCOL

Observational Study of Individual or Group Template

Mindfulness-based Neurofeedback to augment DBT psychotherapy for adults with Borderline Personality Disorder (MIND-BPD)

Protocol Number

2000037582

Protocol Version

July 23, 2025

Version # 8

Synopsis

**Purpose**<br>The overall purpose of this study is to test the ability of mindfulness-based real time fMRI neurofeedback (mbNF) to increase the benefits of evidence-based psychotherapy for adults with Borderline Personality Disorder (BPD).
**Primary Objective**<br><br>The primary objective of this study is to determine whether mbNF (compared to control NF) improves response to psychotherapy in adults with BPD, and if the mechanism of mbNF action is by changing resting state functional connectivity of the default mode network (DMN).
**Secondary Objective**<br><br>The secondary objectives of this study are:<br>To explore whether mbNF (compared to control NF) increases mindfulness in adults with BPD<br>To explore whether mbNF-mediated neural and symptom changes persist after the end of mbNF and psychotherapy.
**Study Design**<br>This is a prospective randomized double-blinded parallel group study testing the impact of mbNF compared to controlNF on psychotherapy response in adults with BPD. Participants will be randomized to one session of NF (mbNF or controlNF), then after completing the NF, all participants will participate in remote once-weekly sessions of psychotherapy (Dialectical Behavior Therapy skills groups) for 20 weeks.<br>Participant eligibility will be determined by clinical interviews and surveys.<br>Outcome measures will include data collected from fMRI and from surveys. Participants may also be asked to participate in focus groups **to discuss** their experiences with the study interventions.
**Study Date Range and Duration**<br>We anticipate that the study will last ~ 9 months for each participant, including ~ 1 month screening and eligibility determination, ~ 1 month to schedule and complete the NF session, 5 months of psychotherapy, and 2 months of follow-up.<br>Participants will be asked for permission to re-contact them after the end of the study for later follow-up.

**Number of Study Sites**<br><br>Three: Yale University, at The Athinoula A. Martinos Center for Biomedical Imaging at Massachusetts General Hospital (MGH), and University of Massachusetts Amherst.
**Primary Outcome Variables**<br><br>1. **Response to psychotherapy** will be measured by change in BPD symptom severity as measured by the "Borderline Symptom List 23-item" (BSL23) survey.<br>**2. Change in resting state functional connectivity of the DMN** will be measured by testing the resting state fMRI data for:<br>a) within-DMN connectivity (defined by connectivity between core nodes of medial prefrontal cortex (mPFC) and posterior cingulate cortex) and<br>b) DMN-FPCN anticorrelation (defined by connectivity from mPFC to dorsolateral prefrontal cortex (dlPFC))
**Secondary and Exploratory Outcome Variables**<br><br>1) Mindfulness will be measured by the State Mindfulness Scale<br>2) Whether neural and clinical changes persist will be measured at follow-up timepoints using the primary outcome variables
**Study Population**<br><br>Adults with Borderline Personality Disorder (BPD)
**Number of Participants**<br><br>124 completers, as well as up to 20 mbNF pilot participants (10 in New Haven, 10 in Boston) (if participants dropout mid-study, we may replace them)
**Study Schedule**<br>1. Screening visits (3-6 hours, usually remote) include the consent process, baseline clinical measures, and eligibility decision.<br>2. Neurofeedback session (~ 3 hours) includes arrival, final safety checks, mindfulness training, functional MRI scans and neurofeedback in MRI scanner, and debriefing procedures.

3. DBT skills group psychotherapy: 60-120 minutes weekly for 20 weeks, additional surveys to complete ~ every 5 weeks.
4. Repeat MRI scans at DBT week 16, and 2 months after DBT ends are each ~ 90 minutes.
5. Some participants may be asked to participate in focus groups about the acceptability of the intervention.

Protocol Revision History

Version Date	Summary of Substantial Changes
V2, 4-23-24	Updated in response to IRB reviewer queries and requests
V3, 8-06-24	Updated to reflect a change in study sites
V4, 10-03-24	Updated to note number of pilot participants
V5, 04-20-25	Updated to reflect additional measures, and revised compensation.
V6, 05-06-25	DSMB approval to start filed along with suggestions from data safety and monitoring board review and requests from Yale IRB to further detail the severe depression response. Assessments added. Revised to greater than minimal risk; annual review added.

Statement of Compliance

This document is a protocol for a human research study. The purpose of this protocol is to ensure that this study is to be conducted according to the Common Rule at 45CFR46 (human subjects) and other applicable government regulations and Institutional research policies and procedures.

Abbreviations

Abbreviation	Explanation
**BPD**	Borderline Personality Disorder
**DBTsg**	Dialectical Behavior Therapy skills group
**fMRI**	Functional Magnetic Resonance Imaging
**mbNF**	Mindfulness based neurofeedback
**NF**	Real time fMRI neurofeedback

Table of Contents

Synopsis 2
Purpose 2
Primary Objective 2
Secondary Objective 2
Study Design 2
Study Date Range and Duration 3
Number of Study Sites 3
Primary Outcome Variables 3
Secondary and Exploratory Outcome Variables 3
Number of Participants 4
Study Schedule 4
Protocol Revision History 5
Statement of Compliance 6
Abbreviations 7
Table of Contents 8
1 Background/Literature Review 11
1.1 Background 11
1.2 Prior Experience 14
2 Rationale/Significance 15
2.1 Rationale and Study Significance 15
2.2 Purpose of Study/Potential Impact 15
2.3 Potential Risks and Benefits 15
2.3.1 Potential Risks 16
2.3.2 Potential Benefits 17
3 Study Purpose and Objectives 17
3.1 Hypothesis 17
3.2 Primary Objective 18
3.3 Secondary Objective 18
4 Study Design 18

4.1.1 General Design Description 18

4.1.2 Study Date Range and Duration 18

4.1.3 Number of Study Sites 19

4.2 Outcome Variables 19

4.2.1 Primary Outcome Variables 19

4.2.2 Secondary and Exploratory Outcome Variables 20

4.3 Study Population 20

4.3.1 Number of Participants 20

4.3.2 Eligibility Criteria/Vulnerable Populations 20

5 Study Methods/Procedures 21

5.1 Study Procedures 22

5.1.1 Data Collection 32

5.1.2 Adverse Events Definition and Reporting 32

5.2 Study Schedule 35

5.3 Informed Consent 35

5.3.1 Screening Procedures 35

See section 5.1 35

5.3.2 Recruitment, Enrollment and Retention 35

5.4 Statistical Method 36

5.4.1 Statistical Design, Sample size determination, and Planned Analyses....................36

5.4.2 Analysis of Subject Characteristics 37

5.4.3 Interim Analysis 38

5.4.4 Handling of Missing Data 38

6 Trial Administration 38

6.1 Ethical Considerations: Informed Consent/Assent and HIPAA Authorization 38

6.2 Institutional Review Board (IRB) Review 39

6.3 Subject Confidentiality 39

6.4 Deviations/Unanticipated Problems 40

6.5 Data Quality Assurance 41

Study staff will be trained to good inter-rater reliability for study assessments, and to adherence for mindfulness training and DBT skills group psychotherapy....................41

6.6 Study Records 41

Regulatory documents 41
Protocol 41
Consent forms 41
Interview results 41
Surveys 41
Study visit notes 41
Psychotherapy records..41
MRI data..41
6.7 Access to Source..41
6.8 Data or Specimen Storage/Security 42
Data will be stored using secure electronic storage systems, including..42
6.9 Retention of Records..42
We will store de-identified records indefinitely. We will destroy identifiers within one year after planned analyses are complete. 42
6.10 Study Modification..42
6.11 Study Completion..42
6.12 Funding Source..42
6.13 Publication Plan 42
7 References 43

1 Background/Literature Review

1.1 Background

A.1. BPD is associated with significant burden of disease Borderline Personality Disorder (BPD) is a debilitating mental illness affecting ~6% of the US population.[1] People with BPD have low rates of functional recovery (only 60% in a 16-year follow up study)[2] and early mortality from both medical and psychiatric causes.[3] Suicidal ideation and suicide attempts are common in people with BPD,[4-6] and completed suicide occurs at ~50-fold the rate in the US population.[7] In sum, BPD is common, and carries high risk for lost quality of life, lost productivity, and early death.

A.2. Improved treatments for BPD are urgently needed *A.2.a. Current best practice treatment approach: Psychotherapy for BPD* International guidelines agree that psychotherapy is the main treatment for BPD.[8, 9] Compared to nonspecific control conditions, BPD-focused psychotherapy has moderate clinically relevant effects on overall BPD severity, self-harm, suicidality, and psychosocial functioning.[10] Of the BPD-focused psychotherapies, DBT and Mentalization Based Treatment (MBT) are the most studied, and evidence points to benefits for depression and for psychosocial functioning.[10-12] DBT and MBT have not been compared head-to-head in a randomized trial, but one naturalistic comparison study suggests that DBT might lead to a steeper decline in self-harm and emotion dysregulation than MBT.[13] Traditionally, DBT consists of individual therapy plus skills training across four modules in a group setting. Although most DBT studies have investigated the individual + group combination, a dismantling study demonstrated the importance of the skills training component,[14] and two trials of stand-alone DBT skills group (DBTsg) training had promising outcomes.[15, 16]

A.2.b. Psychotherapy for BPD leaves many patients with unresolved symptoms Though these psychotherapies provide some benefits, drop-out rates are high and many patients do not benefit.[17] Even when receiving these evidence-based treatments, over 40% of completers either remain unchanged or even deteriorate.[18] No medication is FDA-approved for BPD, and evidence suggests that medications may do more harm than good.[19] With the current state of treatment, BPD is associated with immense costs both in terms of individual and family suffering and of health care economics: ~15% of mental healthcare costs go to the care of people with BPD.[20] There is an urgent need for novel approaches to increase efficacy of treatment for BPD.

A.3. fMRI neurofeedback (NF): a new therapeutic tool Numerous studies have demonstrated that fMRI-NF can be used to alter brain function in a targeted manner. For example, a *Science* study used NF to reinforce brain patterns in the visual cortex associated with viewing lines of a specific orientation. After NF, subjects had improved ability to discriminate the targeted line orientation but not other line orientations.[21] The specificity of this effect to the trained orientation is strong evidence that the perceptual effect was induced by the specific brain pattern trained. Importantly, subjects were blinded to the training, so results cannot be attributed to placebo or other nonspecific effects. Other studies have altered facial preferences[22], induced mental associations[23], reduced fear responses[24], and manipulated self-confidence[25] in subjects via fMRI-NF. This body of work illustrates the transformative potential of fMRI-NF to advance human neuroscience by providing a non-invasive tool to test causal relationships between specific aspects of human brain function and hypothesized downstream aspects of mental function.

In addition to the basic science potential of this new technique, there is also great clinical benefit by modulating symptom-relevant brain function. The flexibility of fMRI-NF training to target any aspect of brain function that can be measured in real time during MR imaging creates a rich environment for progressive intervention development. The brain patterns most associated with symptom improvement in each trial can provide alternate targets for future trials that may yield more pronounced clinical improvements. Furthermore, the brain patterns associated with any undesirable mental effects can be identified and trained down

in future trials while, at the same time, desired effects are trained up. Groups around the world, including the two involved in this trial, are now developing fMRI-NF interventions to train patients with neuropsychiatric symptoms toward healthier brain patterns. Early clinical trials have reported promising results across a range of disorders and symptoms, including depression,[26, 27] auditory hallucinations,[28] contamination anxiety,[29] Parkinson's disease,[30] phobia,[24] and Tourette Syndrome.[31] Here we propose to use fMRI-NF to improve mindfulness in BPD patients and thereby amplify their clinical response to DBT.

A.4. Mindfulness as a Mechanism of Change in BPD Treatment Mindfulness refers to the intentional practice of acknowledgment and awareness of the present moment without judgment. Deficits in mindfulness are consistently linked to BPD severity,[32-35] even controlling for emotional and interpersonal dysfunction, underscoring the role of impairments in mindfulness in this disorder. A systematic review of mindfulness training and BPD found that across trials, improvements were found for BPD symptoms.[36] Mindfulness is considered to be the core skill of DBT.[37] Even compared to other DBT modules (i.e. interpersonal effectiveness) in a randomized trial, DBT-mindfulness was associated with decreased impulsivity[38] and BPD symptoms.[39] For DBT patients, mindfulness theoretically facilitates emotional awareness, emotion regulation, and effective rather than impulsive actions[37] Thus, mindfulness *prepares* the subjects to benefit from therapy. Results from several trials support this view. First, mindfulness skills + General Psychiatric Management (GPM, an evidence-based generalist treatment for BPD) vs. GPM alone led to improved clinical outcomes.[40] Second, mindfulness mediated symptom reductions in a trial of DBT in a partial hospital setting.[41] Finally, improvements in mindfulness mediated symptom improvements in DBTsg.[42]

A.5. Default mode network is a neural target relevant to mindfulness that is hyper-engaged in BPD

A.5.a. Default mode connectivity in BPD Previous work has shown that mindfulness is modulated by the functional connectivity of the default mode network (DMN).[43] A meta-analysis of seven large studies in adults with BPD found within-DMN hyperconnectivity in the regions of primary focus for this project (mPFC to PCC).[44] In our preliminary data, we replicate this within-DMN hyperconnectivity and also find decreased anti-correlation between DMN and FPCN in a sample of adults with BPD (***Prelim. Data 1; Fig 3***).

A.5.b. Default mode to amygdala connectivity in BPD In addition to differences in DMN connectivity, a large literature has implicated amygdala hyperactivation in BPD symptomatology[45, 46] and treatment response[47] and therefore amygdala response to any proposed BPD treatment is of particular interest. In previous studies we developed a mindfulness training and NF intervention to amplify mindfulness (mbNF: described below) and could modulate amygdala function. In particular, an eight-week mindfulness course decreased right amygdala connectivity to subgenual ACC and increased right amygdala connectivity to vmPFC during affective picture viewing.[48] Also, exploratory analyses of our recently collected data suggest that mbNF increased right amygdala to prefrontal connectivity in both adults with schizophrenia and adolescents with depression.[48, 49]

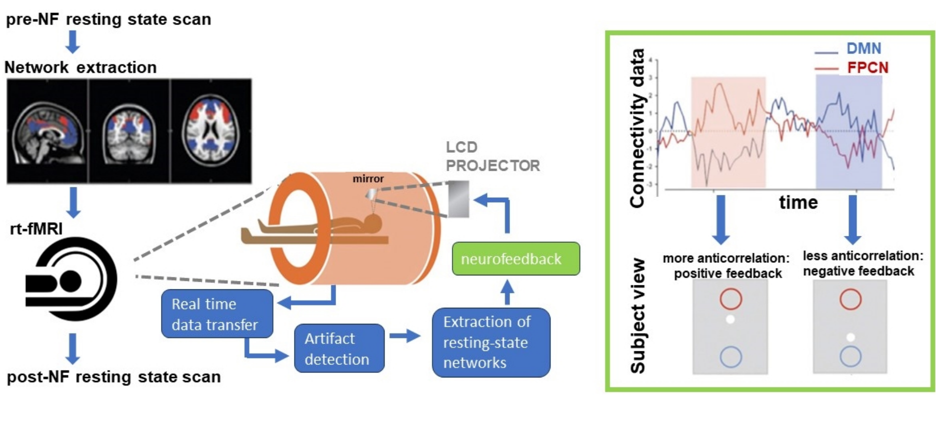

Figure 1. mbNF protocol for network modulation.

A.6. *Mindfulness-based neurofeedback (mbNF) to augment traditional treatment for BPD*

We propose using fMRI-NF to enhance the acquisition and utilization of mindfulness meditation in order to amplify the clinical benefits of DBTsg. We recently developed an innovative NF paradigm to augment mindfulness meditation: people observe a visual display of the difference between real-time DMN and frontoparietal control network (FPCN) activation levels and use mindfulness as a strategy to volitionally regulate this difference (**Fig. 1**). Our approach is a) personalized to the individual's brain patterns: we use state-of-the-art, individual-level functional brain network mapping techniques to identify personalized neural targets for mbNF[50], b) adaptive: the difficulty is adjusted for each individual both within and between NF runs to ensure optimal learning, and c) demonstrated effective for adjusting DMN connectivity, increasing mindfulness, and improving symptoms in a trans-diagnostic fashion (***Prelim. Data 2,3***).[51] By providing mbNF before DBT, we aim to amplify patients' ability to engage in a mindful state and thereby to learn more effectively from the DBT (**Fig. 2**). Impacts will be personalized: mbNF will position people to master the DBT skills they most need, which likely differ across subjects depending on their specific symptoms.

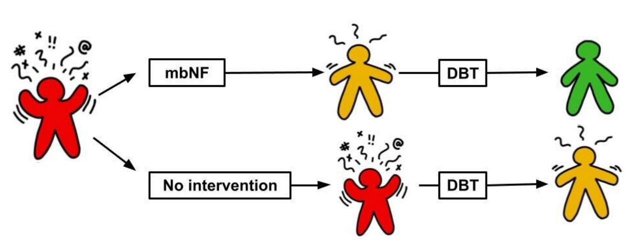

Figure 2. Proposed mechanism of mbNF + DBTsg action. Many people with BPD are not in a state of readiness to fully benefit from DBT. DBT sessions use talk-based approaches to shift patients toward a learning mindset; we expect to accelerate this shift with direct neural modulation before patients begin DBT. We predict that mbNF will alter within-DMN and DMN-FPCN connectivity, and thereby predispose patients toward a more mindful brain state. Engagement of these neural targets is the hypothesized mechanism by which mbNF will develop DBT readiness (not the mechanism of DBT action).

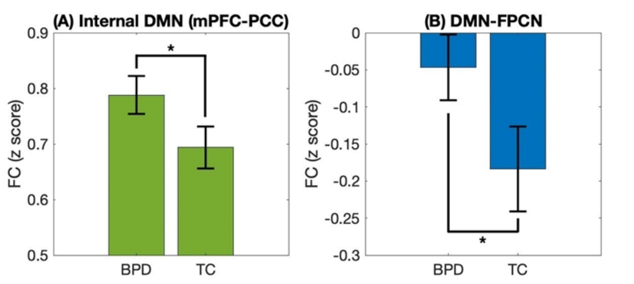

Figure 3. Adults with BPD have DMN connectivity patterns consistent with less mindful brain states vs. healthy controls. In BPD vs. HC, internal DMN connectivity was higher (A) and DMN-FPCN anticorrelation was lower (B) during resting state fMRI. (Unpublished data, one-tailed t-test; error bars are std. error)

A.7. Preliminary Studies and Pilot Data by the Research Team

Preliminary Data 1: We analyzed within-DMN and DMN-FPCN resting state connectivity in 30 adults with BPD and 25 controls recently scanned by the Fineberg lab (**Fig. 3**). Using the proposed methods for this study, we found greater within-DMN (mPFC to PCC) connectivity in BPD than control (d=0.50, $t(1,53)$=1.84, p=.04; z-scores: Mean HC 0.69, Mean BPD 0.79). This suggests hyper-connectivity in BPD vs HC, consistent with the idea that BPD is having brain activity more consistent with mind-wandering. We also found DMN to FPCN (defined as dlPFC) connectivity to be less anticorrelated in BPD than HC (d=0.52, $t(1,53)$=1.93, p=.03; z-scores: Mean HC -0.18, Mean BPD -0.05). This suggests that the expected DMN-FPCN anti-correlation is present

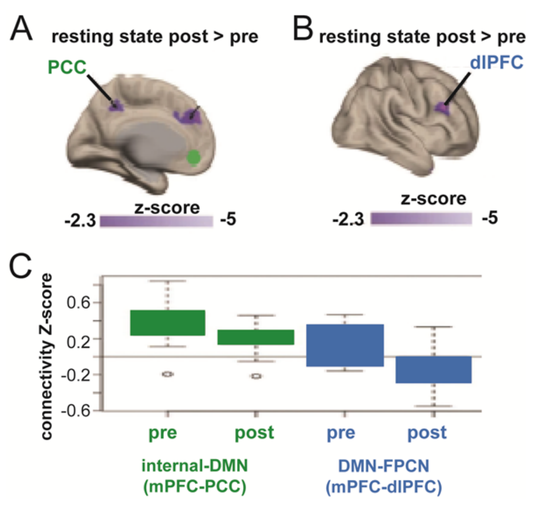

Figure 4. mbNF reduced internal DMN connectivity (A,C green bars) and increased DMN-FPCN anticorrelation (B,C blue bars) during resting state fMRI in schizophrenia patients (n=11). Green dot is mPFC seed. Adapted from Bauer 2020.

in HC, and weaker in BPD, consistent with less of a mindfulness brain activity pattern in BPD than in HC.

- ***Preliminary Data 2:*** **mbNF reduces within-DMN connectivity and increases DMN-FPCN anticorrelations in patients diagnosed with schizophrenia.** In our initial study testing mbNF in 11 schizophrenia patients,[28] it reduced within-DMN connectivity (**Fig 4A, C** shows decrease in mPFC-PCC connectivity from pre- to post-mbNF, Cohen's d=.98, p<.05) and increased DMN-FPCN anticorrelation (**Fig 4B, C** shows increased anticorrelation mPFC-dlPFC, d=.74, p<.05). Patients also had reduced symptom scores (lower auditory hallucination scale scores) after mbNF but not after control feedback of motor cortical activity. We replicated this finding in a more recent study in which we again compared mbNF to motor cortical NF. Compared to control NF (n=13), mbNF (n=12) exhibited a significantly greater reduction of within-DMN connectivity (d=0.71, p<.05, unpublished data).
- ***Preliminary Data 3:*** **mbNF leads to within-person DMN connectivity change in depressed adolescents**. In line with our proposed study, mbNF reduced within-DMN connectivity (mPFC-PCC; d=2.05, p<.001) in each of 9 depressed adolescents.[26] Further, as expected, after mbNF, subjects had increased state mindfulness (d=.63, p<.05, one-tailed) that was positively correlated with time spent in target brain state during mbNF training. Critically, during post-mbNF evaluation, no subject reported headache, fatigue, or pain, and all of the subjects completed the mbNF and resting state procedures. These pilot data highlight the feasibility and tolerability of mbNF, and critically, demonstrate target engagement. This R61/R33 will now extend this work to adults with BPD and test whether mbNF augments effects of BPD-focused psychotherapy.

1.2 Prior Experience

- ***Preliminary Data 4: Feasibility of NF for adults with BPD*** fMRI-NF is feasible in BPD. Paret et al.[52] demonstrated that over four NF sessions, 8 women with BPD successfully downregulated their amygdala activation when cued to do so in an amygdala-focused NF paradigm. This intervention also led to increases in functional connectivity between amygdala and prefrontal cortex. In a second study testing the same intervention in twenty-five women with BPD, improvements were again observed in self-report measures of BPD symptom severity, affective lability, emotion regulation, and a psycho-physiologic measure of startle response.[53] However, we are not aware of any published data demonstrating benefit of NF in a controlled trial for BPD. In pilot work at Yale, we have demonstrated feasibility to conduct NF with adult BPD research subjects. Three pilot subjects were recruited and successfully completed a multi-session NF training.
- ***Preliminary Data 5: Feasibility of remote DBT for adults with BPD*** We have also demonstrated feasibility of our planned psychotherapy approach. Online delivery of DBT is thought to be feasible, acceptable, and safe, with similar clinical improvements and increased attendance compared to in-person DBT (reviewed in[54]). Satisfaction is high,[55] clinicians view this approach positively.[56] Although additional attention is needed to manage risk, best practice guidelines are available to guide decision-making.[57, 58] These published data fit with our experience: Dr. Dixon-Gordon directs an active remote DBT skills group through the UMass Psychological Services Center with weekly groups running according to the model that we plan for this grant. She now has several years' experience with risk management in remote DBTsg. Two of our pilot NF subjects also enrolled in Dr. Dixon-Gordon's remote DBTsg program. They reported that the group was very useful and they attended most scheduled sessions. Taken together, these data demonstrate feasibility for our team to provide both the NF and DBTsg in the planned study.

2 Rationale/Significance

2.1 Rationale and Study Significance

As described in the background sections above, BPD is a complex disorder for which available treatments are sorely lacking in efficacy, efficiency, and scalability. mbNF has shown benefits in other disorders (depression, schizophrenia). The proposed mechanism of action of mbNF is increase in the mindful brain state. This is a promising approach to augmentation for DBT, an evidence-based psychotherapy for BPD for which mindfulness training is a core focus. MIND-BPD will provide novel data to speak to the potential benefit of combining mbNF with DBTsg. DBT-based approaches have been used to benefit people with a wide range of psychiatric disorders. This project will also potentially lay the groundwork for future directions to support the combination of mbNF with other disorder-specific psychotherapies.

Furthermore, ongoing work suggests that mbNF is useful for a wide range of mental health problems (we already have data as well as current projects in depression and psychotic disorders) and age groups (we have data in adolescents and adults), and that it may be able to be implemented in the future with cheaper EEG-based approaches, which will be important if it turns out to be effective.

2.2 Purpose of Study/Potential Impact

The main goal of this study is to test the utility of mbNF + DBTsg for adults with BPD, and to determine the role of DMN connectivity as a mechanism of action.

However, even if no changes in the target mechanism are found specific to the mbNF group, one feature of this study is that we will collect fMRI data and mindfulness and clinical measures before, during, and after DBT, and this will allow us to track changes in brain activity and associated changes in mental function as the treatment unfolds. For example, we will be able to test if mindfulness and mindfulness-associated brain states mediate clinical response to DBT, regardless of whether mbNF outperforms controlNF. We will also be able to explore whether alternative neural circuitry is primarily responsible for clinical response using whole-brain resting-state connectivity analyses to investigate alternative mechanisms. In short, regardless of the outcome the study will provide a rich source of clinically relevant information.

2.3 Potential Risks and Benefits

2.3.1 Potential Risks

The questionnaires and tasks used in the current study should not pose any lasting risk to the well-being of the participants. Participants in this study may experience temporary feelings of distress due to the nature of the questions asked by the experimenter and in the surveys they complete, as well as frustration during some of the tasks.

Magnetic resonance (MR) is a technique that uses magnetism and radio waves, not xrays, to take pictures and measure chemicals of different parts of the body. The United States Food and Drug Administration (FDA) has set guidelines for magnet strength and exposure to radio waves, and we carefully observe those guidelines. Participants will be watched closely throughout the MR study. Some people may feel uncomfortable or anxious. If this happens to any participant, they may ask to stop the study at any time and we will take them out of the MR scanner. On rare occasions, some people might feel dizzy, get an upset stomach, have a metallic taste or feel tingling sensations or muscle twitches. These sensations usually go away quickly but participants will be asked to tell the research staff if they have them. The MR scanning sessions also require participants lying on your back in the scanner, which can become uncomfortable. You will be wearing head gear that will enable you to communicate with study staff during the scanning sessions. If you develop back pain or any other problems you should let them know. There are some risks with an MR study for certain people. If participants have a pacemaker or some metal objects inside their body, they may not be in this study because the strong magnets in the MR scanner might harm them. The MR scanner is also very loud. For comfort and to protect hearing, participants are given ear plugs before entering the scanner. It is important that these are inserted correctly and are effectively dampening noise before scanning begins. You will still be able to hear the study staff over your head phones while in the scanner despite wearing these ear plugs.

The MR scanner creates very strong magnetic fields. Thus, another risk of MR scanning is the possibility of metal objects being pulled towards the magnet and hitting them. To lower this risk, all people involved with the study must remove all metal from their clothing and all metal objects from their pockets. We also ask all people involved with the study to walk through a detector designed to detect metal objects. It is important to know that no metal can be brought into the scanner room at any time. Also, once participants are in the scanner, the door to the room will be closed so that no one from outside accidentally goes near the scanner. **We want participants to read and answer very carefully the questions on the MR Safety Questionnaire related to their personal safety**. We will provide all participants with time to be sure that they have read the MR Safety Questionnaire and be sure to tell us any information they think might be important.

This MR study is for research purposes only and is not in any way a complete health care imaging examination. The scans performed in this study are not designed to find abnormalities. The principal investigator, the lab, the MR technologist, and the Magnetic Resonance Research Center are not qualified to interpret the MR scans and are not responsible for providing a health care evaluation of the images. If a worrisome finding is

seen on any participant's scan, a radiologist or another physician will be asked to review the relevant images. Based on his or her recommendation (if any), the principal investigator or consulting physician will contact them, inform them of the finding, and recommend that they seek medical advice as a precautionary measure. The decision for additional examination or treatment would lie only with the participant and their physician. The investigators, the consulting physician, the Magnetic Resonance Research Center, and Yale University are not responsible for any examination or treatment that they receive based on these findings. The images collected in this study are not a health care MR.

We anticipate minimal risks to participant privacy given the procedures to protect confidentiality described below.

Participants may find participation in DBTsg to be temporarily distressing due to the emotions and situations discussed. We have specific plans to prepare for and mitigate risk (see below).

2.3.2 Potential Benefits

Participants in the study may find that participating in evidence-based psychotherapy for BPD (DBTsg) helps them feel better.

Participants may benefit from increased mindfulness after mindfulness training and neurofeedback on the mbNF day.

Some participants report benefiting from contributing to research that may help others in the future.

Some participants report that the clinical assessments are enlightening as they provide a structured way of describing and thinking about their symptoms and life experiences.

We are conducting this study with the aim of identifying a new treatment option for adults with BPD, which would benefit the BPD population and their loved ones and would reduce BPD-associated healthcare costs to society in general.

3 Study Purpose and Objectives

3.1 Hypothesis

mbNF (compared to control NF) improves response to DBTsg psychotherapy in adults with BPD, and it does so by shifting resting state functional connectivity of the DMN toward a move mindful brain state (reduced within-DMN connectivity, and increased frontoparietal / DMN anti-correlation).

3.2 Primary Objective

The primary objective of this study is to determine whether mbNF (compared to control NF) improves response to psychotherapy in adults with BPD, and if the mechanism of mbNF action is by changing resting state functional connectivity of the default mode network (DMN).

3.3 Secondary Objective

The secondary objectives of this study are:

1) To explore whether mbNF (compared to control NF) increases mindfulness in adults with BPD
2) To explore whether mbNF-mediated neural and symptom changes persist after the end of mbNF and psychotherapy.

4 Study Design

4.1.1 General Design Description

This is a prospective randomized double-blinded parallel group study testing the impact of mbNF compared to controlNF on psychotherapy response in adults with BPD. Participants will be randomized to one session of NF (mbNF or controlNF), then after completing the NF, all participants will participate in remote once-weekly sessions of psychotherapy (Dialectical Behavior Therapy skills groups) for 20 weeks.

Participant eligibility will be determined by clinical interviews and surveys.

Outcome measures will include data collected from fMRI and from surveys. Participants may also be asked to participate in focus groups about their experiences with the study interventions.

4.1.2 Study Date Range and Duration

We anticipate that the study will last ~ 9 months for each participant, including ~ 1 month screening and eligibility determination, ~ 1 month to schedule and complete the NF session, 5 months of psychotherapy, and 2 months of follow-up.

Participants will be asked for permission to re-contact them after the end of the study for later follow-up.

4.1.3 Number of Study Sites

Three: Yale University, MGH, and University of Massachusetts Amherst. Consent will only take place at Yale.

Yale activities: all phone screens, all recruitment, consent, and baseline and follow-up assessments (usually all remotely by zoom). MRI scans, including NF scans for the participants local to the New Haven area and scans for pilot participants.

MGH activities: MRI scans, including NF scans for the participants local to the Boston area and scans for pilot participants.

U Mass: remote psychotherapy group for all participants.

4.2 Outcome Variables

4.2.1 Primary Outcome Variables

1. Response to psychotherapy will be measured by change in BPD symptom severity as measured by the "Borderline Symptom List 23-item" (BSL23) survey. BSL23 is a 23-item scale that measures BPD symptom severity over the past one week. It has established reliability (Cronbach's α = 0.94-0.96) and validity (r = 0.96 versus the longer BSL-95, r = 0.87 versus the Beck Depression Inventory, and r = 0.48 versus the general psychopathology scale SCL-90) in initial psychometric studies.59 It is sensitive to clinical change (d=0.47 from before to after DBT).59

2. Change in resting state functional connectivity of the DMN will be measured by testing the resting state fMRI data for:

a) within-DMN connectivity (defined by connectivity between core nodes of medial prefrontal cortex (mPFC) and posterior cingulate cortex), and
b) DMN-FPCN anticorrelation (defined by connectivity from mPFC to dorsolateral prefrontal cortex (dlPFC))

Four seed connectivity maps will be created for each subject (pre-NF, post-NF, DBT week 16 & follow-up) using an 8mm spherical mPFC seed region centered on the mPFC DMN locus from Fox, *et al.*, 2005.[60] A composite of the baseline (i.e., pre-NF) seed maps across subjects will then be used to identify two small-volume corrected regions of interest (ROIs): a positive locus in the PCC and a negative locus in the dlPFC (CONN toolbox definitions of the PCC and dlPFC will provide the anatomical regions within which these are identified). The mean value in the mPFC seed map for each subject within the PCC ROI will provide a measure of the within-DMN connectivity for that subject at that timepoint. Similarly, the mean value within the dlPFC will provide a measure of their DMN-FPCN connectivity.

4.2.2 Secondary and Exploratory Outcome Variables

1. Mindfulness will be measured by the State Mindfulness Scale, a 21-item self-report measure that quantifies current-moment mindfulness of body and mind.61
2. Whether neural and clinical changes persist will be measured at follow-up time points using the primary outcome variables

4.3 Study Population

The study population is adults with BPD. We will recruit primarily from social media, targeting people living in the New Haven and Boston metro areas.

4.3.1 Number of Participants

124 completers, plus up to 20 additional mbNF pilot participants (10 in New Haven, 10 in Boston), who will not undergo psychotherapy. (If participants dropout mid-study, we may replace them).

4.3.2 Eligibility Criteria/Vulnerable Populations

As above, we plan for 124 people to complete the overall study. We expect that enrollment will be balanced between the New Haven and Boston cohorts, for a total of 62 participants per site. This means that we expect a final total of 31 completers randomized to each group (mbNF and control NF) at each site at the end of the study.

This study is open to people of any sex at birth and any gender.

Inclusion criteria. All subjects must: be age 18-60, be able to provide written informed consent, meet criteria for BPD on semi-structured clinical interview, be able to plan to keep any prescribed medications and psychotherapy constant during the study, and be fluent in English.

Exclusion criteria. Potential subjects will be excluded for:

- current DBT psychotherapy outside the study
- lifetime primary psychotic disorder or Bipolar I disorder
- developmental disorder (e.g. autism)
- history of learning disorder
- moderate or severe substance use disorder in the last 6 months
- active suicidal ideation with intent or plan in the past 3 months
- history of major medical or neurologic disorder
- MRI contraindications, including pregnancy
- poor performance on reading task (WRAT > 11 errors)
- newly prescribed medications in the past 8 weeks
- daytime sedating medications (e.g. benzodiazepines, opiates, sedating neuroleptics)
- any scheduled daily benzodiazepines
- change in psychotherapy type or frequency in the past 12 weeks.
- At the discretion of the study PI

Termination criteria.

- Enrolled participants will be terminated from DBT group participation for:
  - Missing over 3 consecutive DBT group sessions. These participants will still be invited to complete study assessments.
- Enrolled participants may be terminated from the study:
  - At the discretion of the study PI

Eligibility will be determined by study personnel.

Medication considerations. Medication use is common in our target sample. Though adding some complexity, allowing medication will increase representativeness and generalizability. We will require that study participants not be taking daytime sedating medications. Treatment prior to and during the study will be recorded and explored in analyses. We will code medications into 5 classes: antidepressants, antipsychotics, mood stabilizers, anxiolytics, stimulants. We check if overall binary medication variables (psychotropic medication or not) or class-level medication binary variables (presence/absence of each medication class) differ between groups and if so, we will include them as covariates in the statistical analyses.

Vulnerable populations

We will not include people who are pregnant or trying to become pregnant. We will not specifically recruit people from disadvantaged groups or people with impaired decision-making capacity for this study.

5 Study Methods/Procedures

5.1 Study Procedures

Screening and Eligibility Determination

Phone screens will briefly query demographics, psychiatric and medical diagnoses, BPD symptoms, medications, interest in a longitudinal study, and MRI safety. Potentially eligible subjects will be invited to a screening visit where trained interviewers assess inclusion and exclusion criteria using semi-structured instruments: Diagnostic Interview for Personality Disorders BPD section[62], Structured Clinical Interview for DSM5 for general psychopathology.[63]

Clinical Baseline and Follow-up Assessments

Clinical and demographic measures will be collected at baseline, before and after the neurofeedback day, at the start of each DBT module, and at each of the two follow-up timepoints.

Demographic data (including age, years of education, ethnicity, occupation, hours per week working, and relationship status) as well as medical history, psychiatric history, current medication list, and adherence will be collected from all participants who give consent. Should we decide to, in order to pursue specific questions of task performance in specific groups, we will use these screens to enroll specific balanced groups of people will specific demographics, such as limited age range, gender, or medications.

All enrolled participants will complete several psychological questionnaires.

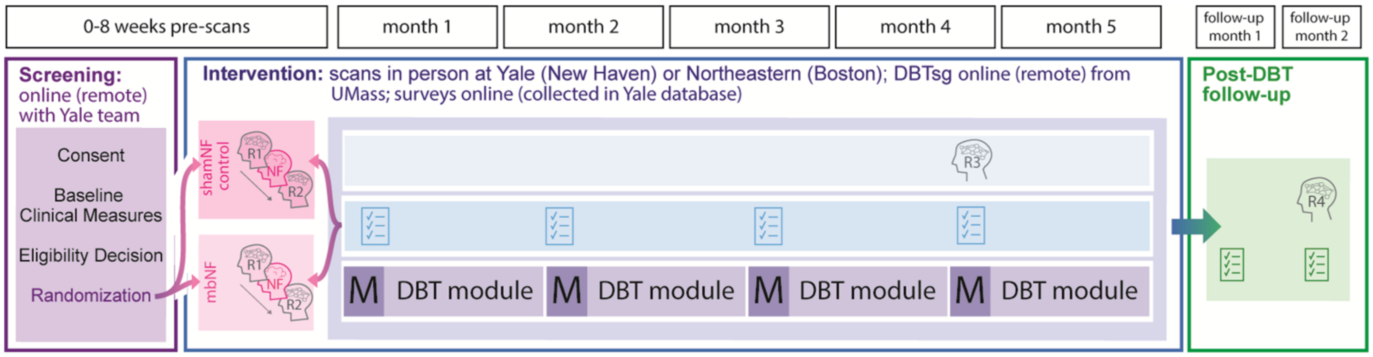

Figure 6. Overview of study subject activities Each subject is screened and, if eligible, receives 5.5 months of intervention then 2 months of follow-up (timeline at top). All clinical assessments in the **Screening**, **Intervention**, and **Follow-up** periods are done by the Yale team by remote video-conference.

- **Screening period**:
  1. Subjects consent, then clinical assessments are used to determine eligibility and collect baseline data.
  2. Eligible subjects are then randomized to **mbNF** (the active neurofeedback intervention) or **shamNF** (the yoked-sham control intervention).
- **Intervention period:**
  1. Neuroimaging day 1 before scan: baseline mindfulness / clinical symptom surveys, mental noting mindfulness training outside the scanner
  2. fMRI scan to collect data for personalized functional localization and baseline measurements of connectivity ( ).
  3. Neurofeedback ( ) ,mbNF or shamNF, subject and assessors are blind to condtion).
  4. Post NF resting scan fMRI scan ( ) for R61 primary outcome of target engagement. Mindfulness/clinical symptoms are assessed one week post-NF
  5. DBT skills group start 1-2 weeks after NF: four 5-week blocks, each with 1 week focused on mindfulness (M) then a 4-week DBT module.
  6. On each mindfulness day (M): surveys ( ) on past-week mindfulness/clinical symptoms.
  7. Primary study endpoint (DBTsg week 16): resting state fMRI scan ( ), clinical/mindfulness surveys
- **Follow-up period:** clinical assessments at follow-up months 1 and 2, final resting state fMRI scan at follow-up month 2 ( ).

Table 1. Psychological questionnaires

Domain	Questionnaire	Expected time
Psychiatric diagnosis	Review of psychiatric history, and major life events, including educational history, family history, and major mental health events<br>*“Thinking about your adult life, what are the major events that have occurred in your life, in your family, in your schooling, and in your mental health?”*<br>SCID I psychosis, mania, depression, substance use disorder, and alcohol use disorder	1 hour
Borderline Personality Disorder diagnosis*<br>*note: if a potential participant meets criteria for BPD and does not already carry a diagnosis, a clinical staff member involved with the study will discuss the BPD diagnosis and conduct a diagnostic disclosure.	DIPD-BPD	30 mins
Current and past suicidal ideation and self-injurious behaviors	Columbia Suicide Severity Scale (CSSRS)	30 mins
Borderline Personality Disorder severity	BSL-23	5 min
State Mindfulness Scale	SMS	5 min
Suicidal Thinking	BSS	5 min

Suicidality and Imminent Risk	University of Washington Risk Assessment Protocol (UWRAP)	10-30min
Trauma history	Life Events Checklist (LEC-5)	5 min
Depression	BDI	5min
Emotion regulation	DERS	5 min
IQ, English reading ability	WRAT	2 min
Dissociation	DES	5 min
State PTSD symptoms	PCL-5	5 min
Social functioning	Social Adjustment Scale – Self report	5 min
Picture Description	Participants are asked to describe aloud complex images (e.g. photographed scenes) for 3 minutes to capture their natural narrative style.	3 min
Future Thinking	Participants are asked to describe a realistic future event that could happen in their life in the next 5 years	3 min
Non-Suicidal Self-Injurious Behaviors	SITBI-R (revised)	5 min
NIMH-mandated assessment of depressive symptoms	PHQ-9	2min

NIMH-mandated assessment of anxiety symptoms	GAD-7	2min
NIMH-mandated assessment of psychosocial wellbeing	WHODAS	5min
NIMH-mandated screener for DSM disorders	DSM-5 Level 1 Cross-Cutting Symptom Measure (CCSM)	5min
Trait Mindfulness	MAAS	2min
Handedness	Edinburgh Handedness Inventory (EHI)	1min
Participant engagement in intervention	Intrinsic Motivation Inventory (IMI)	5min
State Dissociation	Dissociative Symptoms Scale, 4-item version (DSS-4)	2min
Participant sense of emotional and bodily agency	Self of agency scale	3 min
Interpersonal Emotion Regulation	Difficulties in Interpersonal Emotion Regulation (DIRE)	5 min
Emotional invalidation	Perceived Invalidation of Emotion Scale	2 min
Shame	Shame Scale – 3 item	1 min
Personal and clinical recovery and hope for recovery	Recovery Assessment Scale (RAS)	4 min

Evaluation of how study participants feel about the way we go about assessing and responding to perceived risk of harm to self and others in the study	Qualitative Interview to assess risk assessment procedures	10 min
Evaluation of social networks	Social network survey	~ 15 min

Psychological Tasks (= computer-based activities)

Self-reference task	Participants are asked to respond to prompts on the computer screen during fMRI scanning about the personality attributes of themselves, close friends, and strangers by pressing buttons to indicate yes, no, or how much a statement is true.	~ 15 min
Transitions task	Participants are asked to respond to questions about their own and other people's mood and changes in mood. This task is done outside of the scanner.	~20 min

Safety / Risk Assessment

Oversight of risk assessment and response

Overall risk will be managed by Dr. Fineberg in consultation with Dr. Dixon-Gordon (site PI at U Mass), and the MGH study clinician. The study team will meet regularly (at least every two weeks) to review recruitment and risk management.

Risk assessment plans built into scheduled study procedures

- Safety plans: Each patient will work with study staff to complete a safety plan at the beginning of the study with either clinical assessment team or DBT team using a standard approach (Brown and Stanley method). This safety plan includes specific plans for thoughts and behaviors to do if risk level is low, moderate, or at crisis level. For example, something to do if risk is low could be to practice mindfulness meditation. Something to do if risk is moderate could be to leave an upsetting situation. Something to do if risk is at crisis level could be to call 988 for support or call a helpful friend to come over to provide support. The plan includes specific contact details for resources, including both those suggested by the participant during the safety planning conversation that results in the written plan, and also including a standard set of local and national resources included in every plan (988, crisis text line, local resources for New Haven or Boston depending on the participant's location). Once made, the plan resides in REDCap to help study staff support participants as needed throughout the study, and is emailed to the participant right away, and again as needed to allow them to use it and to share it with their support system. Participants are encouraged to view the safety plan as a living document and to ask study staff to update it anytime. We encourage participants to keep a paper copy with them (in their wallet or similar). If participants don't have easy access to a printer, study staff will offer to send it in the mail or bring it to the scanning day, depending on participant preference.

- Risk assessment tool 1: UWRAP In addition to the safety plan, the risk assessment tool (UWRAP) will be introduced at baseline to help participants become familiar with it and to assess baseline risk. This tool assesses distressing emotions and urges for dangerous behaviors on a 7 point Likert scale. At the end of each clinical assessment timepoint, as well as on an as-needed basis throughout the study, risk will be re-assessed with the UWRAP tool. If a participant endorses a 4 or greater for any item, further discussion of the situation will occur, and the participant will be offered a number of supportive activities from the UWRAP list and based on their personalized safety plan. Then risk will be assessed again using the scale.

- Risk assessment tool 2: CSSRS We will also assess suicidal ideation and behavior formally at each clinical assessment timepoint (baseline, after NF, at the start of each DBT mindfulness module, and at follow-up months 1 and 2) using the C-SSRS.

- Risk assessment through staff/participant interaction Fineberg lab staff and DBT program staff may also become aware of subject distress or symptom exacerbation, including suicidal ideation.

Risk assessment by clinical study staff when risk is detected

What will trigger study staff to immediately involve a study clinician for further assessment:

- Participant who is unable to participate in making a detailed safety plan
- Participant who endorses 4+ on any UWRAP item and level does not decrease below 4 after using UWRAP-suggested or safety plan-suggested behaviors
- Participant who endorses of type 4 or 5 ideation (suicidal ideation with intent or plan) or new suicidal behavior
- Any study staff concern that the staff member would like support in addressing

What study clinicians will do when contacted about risk:

Each study site has local study clinicians assigned to support risk management. Risks identified in the course of study recruitment (to be conducted by the Yale site staff) or the in-person mbNF day at Yale will be reported to Dr. Fineberg. Risks identified in the course of the in-person mbNF day at MGH will be reported to the on-call study clinician (to be named) during the visit. Risks identified in the course of remote DBT will be reported to Dr. Dixon-Gordon according to standard practice for this clinic. Dr. Dixon-Gordon directs an active remote DBT skills group at UMass with weekly groups running according to the model that we plan for this grant. She now has several years' experience with risk management in remote DBTsg, and is prepared to manage risk. She has an active psychology license in Massachusetts and Connecticut.

Any time that study staff would like clinical support or a participant meets one of the criteria listed above, local clinical study clinician will be immediately contacted to discuss the situation and if needed to assess the participant.

Clinical assessments by study clinicians may include review of the participant's thoughts and behaviors in the context of their clinical history to understand current risk of harm to self or others, collaborative review and use of the personal safety plan with the participant, development of immediate next steps including planning strategies to reduce distress and risk, contacting helpful others (friends/family/mental healthcare providers), and if needed, contacting emergency services for further assessment and potential transfer to the emergency department. If emergency services are needed, clinical staff and the local study clinician will make every effort to keep the participant in the visit until the emergency services arrive to ensure safe transfer to their care.

Please refer to section 5.1.2. below for details about reporting of adverse events.

Randomization and control condition
Subjects assigned to receive NF at each site (Yale and MGH) will be randomized within-site using a predetermined randomization schedule prepared and known only to the site neuroimaging staff (the rest of the study staff will remain blinded). Each schedule will result in 1:1 randomization with the constraint that the number of mbNF cases is always ≥ the number of control-NF cases (necessary to ensure each control subject has a prior mbNF subject to be matched to) and also the constraint that the number of mbNF cases does not exceed the number of control-NF cases by more than three (to keep the two groups reasonably balanced over time). Clinical staff and assessment raters will remain blind to group assignment. Subjects will be debriefed at the end of the study and at that time their group assignment will be disclosed.

Yoked-sham feedback Subjects in the control-NF group will complete the mindfulness training and undergo similar MRI scan procedures. The primary difference in scans is that the control-NF subjects will not receive feedback based on their own brain's activity but will receive feedback based on data extracted from a previously-acquired mbNF session with a different study subject. Thus, the visual display will be independent from the subject's own brain activity in the control-NF group. This ensures that subjects across groups are viewing equivalent stimuli. This yoked sham control approach was chosen for this study because it allows for subject blinding, creates a similarly engaging feedback training experience, and balances positive feedback received across groups. Specifically, a control-NF subject receives feedback indicating they are having the same amount of success as the NF subject they are yoked to. The perception of success can impact motivation because it is easier to stay motivated if the participant feels they are having some success. It is also likely to affect

the magnitude of the placebo effect, because if a participant sees that they are successful in controlling their mindfulness-related brain patterns, they are likely to have a larger placebo effect. Thus, controlling for perception of success is critical.

An alternate control used for some studies involves training subjects to control a different brain region that is unrelated to their symptoms. This approach has the disadvantage that success in controlling the region may be significantly different in the control and NF groups, as some brain areas are more easily controlled than others, leading to different perceptions of success on the task and thus differences across groups in both motivation and placebo effects. That being said, one concern that applies to all sham-controlled studies is that subjects in the control group may notice that the feedback does not track with their emotional state, which could damage the blind. For this reason, we will include a debriefing session at the end of our study when we will ask subjects their beliefs about their group assignment (which intervention they think they received, and how confident they are about their group assignment). Subjects and clinical study staff will be blinded to randomization to ensure that the experience of the two groups is as similar as possible. Overall, by providing maximally similar procedures for the mbNF group and the control-NF group, we aim to account for potential mindfulness practice effects (e.g., learning, utilization) and non-specific scanner effects. At each subject's study completion, we will unblind. We will first debrief the subject, and then tell them if they got mbNF or control. We will monitor their affect and, for any subjects who are upset upon learning their group assignment, clinicians will be available to help assess and support them as needed.

Baseline (and Follow-up) MRI scans

We will collect structural and functional MRI scans for participants at their local site (Yale for New Haven area participants, MGH for Boston area participants). We will collect these scans on neurofeedback day, and again at psychotherapy week 16 and at the two month timepoint after the end of psychotherapy. People capable of pregnancy will be offered a voluntary pregnancy test prior to each scan.

A member of the research team will accompany subject to the MRRC and will stay with the subject for the duration of the MR study.

At both sites, images will be acquired with 3.0 Tesla Siemens scanners equipped with a 64-channel head coil and integrated with our real-time fMRI analysis system.[64] We will instruct subjects to minimize head and body motion during scanning, and will continuously monitor head motion online and provide feedback to subjects between scan runs if they move excessively.

MR imaging of the brain will be done, with or without performing a specific task. We may also complete MR imaging of the brain to record the diffusion tensor data. This data can be used to map out fiber tracks in the brain. We may also complete MR spectroscopy of specific brain areas to determine the chemical composition of those brain areas. This data is relevant to the density of neurotransmitters, for example. We will use a standard single-voxel sequence like STEAM or PRESS, for example, that is within the FDA power guidelines for power deposition.

During MR scans, recordings may be taken of the subjects' pulse (via a pulse-oximeter placed on their finger), respiration (via a pneumatic belt), and galvanic skin response (via sensors on the fingers) that are components of Siemens Physiologic Monitoring Unit. We may also monitor the subject's eye movements and/or pupil dilation using a camera and an eye-tracking system. This equipment is designed to be completely MR compatible. The records of pulse and respiration will be used during data processing to remove physiologic noise from the images.

Minimizing MRI Data Loss We will use an online tool that allows monitoring of structural data quality and immediate re-collection as needed.[65] Framewise Integrated Real-time MRI Monitoring (FIRMM)[66] allows for monitoring of head motion artifacts and a real-time extension of resting state run length to ensure sufficient low-motion data for analysis, which greatly reduces imaging data loss due to movement.

Anatomical image acquisition A high resolution, multi-echo (T1-weighted) structural image will be acquired during study baseline sessions for fMRI co-registration. The pulse sequence parameters will be: TR=2.530 ms, TE1=1.64ms, TE2=3.5ms, TE3=5.36ms, TE4=7.22ms, Flip =7°, field of view = 256cm, 1 mm isotropic resolution, acceleration factor of 2, scan time ~6 minutes.

Functional image acquisition Multiband T2*-weighted images will be acquired during resting state and NF runs with the following parameters: acceleration factor=4, 68 transverse slices, TR=1.2 sec, TE=30 ms, Flip=61°, 2 mm isotropic resolution, field of view=256 mm (optimized in prior NF work).[64] During resting state runs (~10 mins), subjects will view a central fixation cross and will be instructed to let their minds wander. During NF runs (six runs of ~2.5 minutes each), subjects will be prompted to follow the mindfulness instructions.

Neurofeedback Day (mbNF or control NF)

Mental Noting training

After the baseline resting state scan, subjects will learn a "mental noting" mindfulness practice to use during the neurofeedback scans. Trained research staff will provide this semi-structured 45-minute training established in our prior work.[28] Mental noting is a major component of Vipassana (insight mindfulness meditation); key principles include "concentration", "observing sensory experience", "not 'efforting' ", and "contentment".[67] Subjects will be taught to mentally label/note whatever sensation is most prominent in their sensory experience from moment to moment (e.g., seeing, hearing, feeling, thinking). This practice primarily trains the "attention and control" mindfulness components, but also influences "attitude" by recognizing that "one's thoughts and feelings are temporary, objective events in the mind, as opposed to reflections of the self that are necessarily true", sometimes referred to as "decentering".[68,69] Training also will include identifying personal scenarios in which mental noting could be applied, explaining the goal of using these strategies to manage distress in daily experiences. Before the MRI scan, subjects will practice mental noting practice by verbalizing their mental label out loud to the study staff, who will observe the practice and provide feedback as needed. Subjects will then complete a silent practice of mental noting while viewing a simulation of neurofeedback. All subjects will be trained until they feel competent to use mental noting in the scanner. During mbNF (6 runs, 2.5 mins each), subjects will use mental noting with the aim of controlling the visual feedback; specifically, they will try to move the position of the white dot toward the (upper) red circle and away from the (lower) blue circle (**Fig 1**).

Mental Noting task with Neurofeedback

During NF, subjects will receive feedback indicating differential activation between DMN and FPCN. As in our previous studies[64, 70] we will use the multivariate and univariate real-time functional imaging (MURFI) package to deliver continuous neurofeedback.[71] Prior to the pre-mbNF resting state run, a brief (2 frame) fMRI localizer will be acquired. During acquisition of the pre-mbNF resting state, we will register personalized network maps from T1 standard MNI space to fMRI space via a linear transform, and trained staff will manually inspect the quality of alignment. We will implement our established neurofeedback protocol,[28] where continuous visual feedback signifies the level of FPCN-DMN activation difference. We will adapt a real-time fMRI computational pipeline described in detail in our previous work.[64] An incremental general linear model (GLM) will be used to compute moment-to-moment variation in activation voxelwise, while accounting for nuisance sources

(e.g. low-frequency signal drifts) computed online.[64] At each timepoint, the GLM will estimate residual signal (based on measured minus expected voxel intensity) that has two components: BOLD signal fluctuations and unmodeled noise. The residual will be scaled by an estimate of voxel reliability (the average GLM residual over the first 15 frames, considered as baseline). Activation within the FPCN and DMN will each be estimated separately, based on the median of all voxels within each network. A trigger signal will be sent to a stimulus presentation computer to control visual feedback. The visual feedback, based on a subtraction of FPCN minus DMN activation, will consist of a rectangle with one centrally displayed white dot and two circles, including a red hollow circle located above and a blue hollow circle located below the white central dot (**Fig 1**). As the FPCN minus DMN difference increases positively (i.e., in the direction of FPCN activation coupled to DMN deactivation), the central white dot will move upward (toward the red circle). As the FPCN minus DMN difference increases negatively, the central white dot will move downward (toward the blue circle). Subjects will aim to volitionally move the central white dot continuously and consistently toward the red using mindfulness strategies.

Psychotherapy (DBTsg)

DBTsg will be a weekly 2-hour group that subjects join by secure videoconference (e.g. Zoom) through the UMass Psychological Services Center. DBTsg will follow the 20-week McMain protocol which led to improvement in treatment > control patients on emotion regulation, distress tolerance, anger, and suicidal/self-harming behaviors.[16] Each subject cohort will proceed through the McMain-protocol DBT modules: Distress Tolerance (4 sessions including skills for “crisis survival,” “distracting,” and “radical acceptance”), Dialectics (4 sessions, including skills for validation, “walking the middle path,” and increasing positive behaviors), Emotion Regulation (4 sessions, including skills for observing and describing emotions, reducing vulnerability to negative emotions, and “opposite action”), and Interpersonal Effectiveness (4 sessions, including skills for clarifying priorities in interpersonal situations, relationship effectiveness, and maintaining self-respect). Each module is preceded by one week focused on mindfulness. We selected this protocol because it is the longest of the evidence-based skills group protocols, with 5 full months of treatment (approaching the 6-month classical schedule).

The DBT groups for this study will be directed by Dr. Dixon-Gordon, who is a clinical psychologist licensed in CT and MA, certified DBT trainer who routinely supervises doctoral students in DBT. We expect this treatment dose to provide meaningful benefit to patients while also facilitating our study design. Admission to the DBTsg program will be rolling, with new subjects admitted on the mindfulness day at the beginning of each module. DBTsg will run continuously throughout the year so subjects can join at the beginning of any module and graduate once they have participated in four full modules. In this way, we can stagger subjects to facilitate the scheduling of NF scans and complete a full cohort.

All DBTsg zoom sessions will be recorded, and participants must agree to video recording in order to be included in the study.

Before, during, and after psychotherapy, mental healthcare use and needs will be tested as exploratory outcomes:

a) (for during study psychotherapy) DBTsg group attendance (scored by study staff by watching videos)

b) use of scheduled and unscheduled mental healthcare resources outside the study (question included in surveys at each assessment timepoint)

Program Evaluation of Subject Experience

We will use a phased approach to evaluate acceptability of the intervention. We will measure subject experience in the domains of tolerability and satisfaction for each of the study procedures in order to ensure that we develop an intervention that will, if effective, be adopted by the target population.

To assess tolerability, we will consider intervention schedule and physical/emotional adverse events.

For satisfaction assessment, subjects will rate treatment expectations, motivation, and likelihood of future participation. Surveys (e.g. Intrinsic Motivation Inventory (IMI)[72]) will quantify participant experience performing NF. Qualitative approaches will query what subjects enjoyed most, what challenges they faced, and their recommendations for improvement.

There will be three phases of the evaluation: setup and planning, pilot, and implementation.

1.Setup and planning The first six months of the R61 will be focused on project setup and procedure piloting, including mbNF protocol setup at Yale and initial work on the program evaluation plan. Dr. Bauer and Ms. Papa will travel to Yale to for pilot tests. Ms. Papa will experience the full protocol and will observe several test subjects in order to define the program evaluation plan.

2. Pilot phase (1.5 years) Data from the first six months will inform the evaluation plan for the first 52 subjects (the NIH R61 phase of the grant). This phase will include testing of self-report questions and exploratory qualitative components, such as post-NF interviews and intermittent focus groups, allowing us to determine the best approaches to measure acceptance and tolerability of the intervention.

3. I*mplementation of program evaluation (3 years)* The final program evaluation plan will be implemented with the subsequent 72 subjects (R33 phase of the NIH grant). Self-report questions for program evaluation will be added to the surveys at baseline, pre-NF, post-NF, and follow-up timepoints. Research assistants will be trained to collect responses to program evaluation questions during the post-NF debriefing. Ms. Papa may also conduct intermittent focus groups by videoconference with subsets of subjects to further explore areas of particular interest. If between-group differences are identified in measures of subject experience, they may be explored as potential moderators of intervention effects. Data from program evaluation will be critical to design of subsequent studies to further develop this intervention and included in manuscripts describing this project.

5.1.1 Data Collection

All documentation of consent, surveys, and interview assessment for this project will be stored in a Yale-based instance of REDCap. Separate REDCap projects will be used for consent with participant identifiers and main data collection (without identifiers, using an alphanumeric code for each participant to provide a link to the consent project and the MR data).

Clinic notes at U Mass will be maintained in the clinic's electronic medical record, including a copy of the clinical intake summary and safety plan (generated by REDCap reports).

MRI data will be stored without participant identifiers on secure servers.

De-identified data will also be transferred to the National Data Archive at regular intervals in order to comply with the mandates of the National Institutes of Health.

5.1.2 Adverse Events Definition and Reporting

No significant adverse events are expected for this intervention. We will query subjects at every clinical assessment for such events. If any occurs, we will consult with the Data Safety Monitoring Board who will have authority to terminate the study, remove patients from participation, etc.

As stated in background section 1.1, suicidal ideation, suicide attempts, and non-suicidal self-injurious behaviors are common in this population. Therefore, these behaviors are not considered to be unexpected adverse events during the course of this study, unless they are directly attributable to study procedures.

5.2 Study Schedule

1. Screening visits (3-6 hours, divided over multiple (usually 2-3) visits, usually remote) include the consent process, baseline clinical measures, and eligibility decision.
2. Neurofeedback session (~ 3 hours) includes arrival, final safety checks, mindfulness training, functional MRI scans and neurofeedback in MRI scanner, and debriefing procedures.
3. DBT skills group psychotherapy: ~120 minutes weekly for 20 weeks, ~ 30 minutes of surveys to complete ~ every 5 weeks.
4. Repeat MRI scans at DBT week 16, and 2 months after DBT ends are each ~ 90 minutes.
5. Repeat surveys for ~ 30-45 minutes at timepoints 1 and 2 months after DBT ends.
6. Some participants may be asked to participate in focus groups about the acceptability of the intervention.

5.3 Informed Consent

Consent forms describing in detail the study intervention, study procedures, and risks are given to the participant and written documentation of informed consent is required prior to starting intervention/administering study intervention. The following consent materials are submitted with this protocol:

MIND-BPD Compound Authorization Form

5.3.1 Screening Procedures

See section 5.1

5.3.2 Recruitment, Enrollment and Retention

Potential subjects will be identified from community advertisements and referrals, primarily from social media advertisements. If we encounter difficulty with recruitment to meet planned targets, we may engage a professional clinical trial recruitment company, such as Build Clinical, to support our recruitment.

All potential participants from any source will be directed to complete a brief screening survey on a secure online platform (e.g. REDCap) to screen out people with common exclusions (e.g. age, location, current DBT psychotherapy, MRI exclusions).

People who may be eligible after the screening survey will be invited to participate in a 15-20 min phone call with a Fineberg lab staff member to identify further common exclusion criteria, including some questions about clinical symptoms. Questions will include demographics, MRI safety and comfort rule-outs, and clinical symptoms to determine likelihood of meeting BPD criteria at full screen.

We will request a waiver of HIPAA authorization for these first two screening steps (for recruitment only), as subjects will be screened in these two steps before the first meeting when the consent process and signing of consent paperwork occurs.

People who may be eligible after phone screen will be invited to participate in a first full screening visit to be conducted by the Fineberg team (for participants at both sites). Those who agree to schedule a visit will be emailed an e-consent form from REDCap which they can review prior to their visit.

If a potential participant joins a first screening visit (always a live meeting, usually remote, with a Fineberg lab staff member), the first step will be the consent process, including a review of study overview and goals, study procedures, study risks and benefits, confidentiality, economic considerations, and in-person expectations regarding infection control for MRI days. Then the participant will have unlimited time to review the e-consent form and ask questions. Only if they decide to sign and submit the form does the visit proceed.

5.4 Statistical Method

5.4.1 Statistical Design, Sample size determination, and Planned Analyses

Data Preparation Data will be examined for outliers, non-normal distributions, and non-linear associations. Preliminary analyses will examine potential covariates including site, age, sex, clinical variables, and head motion. We will explore the presence of group effects (and time effects when appropriate) across individual covariates or combined factors across multiple covariates using a liberal $p<.05$ uncorrected threshold and include any suprathreshold variable as control covariate in subsequent analyses. Detailed information regarding psychiatric service use (e.g., therapy, medication, unplanned mental health service use) will be assessed to test whether this differentially impacts (or is affected by) change in our neural targets. We will also explore baseline symptoms as a potential covariate in the analyses. Conclusions will primarily be based on effect sizes with 95% confidence intervals rather than statistical significance to maximize reproducibility. Based on our prior studies, we estimate ~30% attrition/data loss. We have planned for adequate initial recruitment to anticipate complete data to include 124 subjects.

1. Target Engagement: DBT with mbNF will change resting-state DMN connectivity toward a more mindful brain state.

We will test if mbNF leads to larger changes than control-NF (medium effect-size d≥0.5) in at least one of our two DMN connectivity targets. We will calculate within-subject change from pre- to post-NF, then use one-tailed t-tests (mbNF vs. controlNF) to test our hypothesis that mbNF will lead to larger change by decreasing within-DMN connectivity and/or increasing DMN-FPCN anticorrelation. This effect size is in line with our prior mbNF research in other patient groups (where we saw effect sizes of d=0.7 to d=0.91).

2. Our primary clinical outcome is change in BPD symptom severity (BSL23 survey score) in the full study sample (62/group). Our secondary outcome is change in current-moment mindfulness (SMS score). For each outcome, we will test the hypothesis that mbNF+DBTsg will decrease score more than control-NF+DBTsg from baseline (pre-NF) to primary endpoint (DBTsg week 16) using a linear mixed regression model with group (between-subject) x time (within-subject) interaction. Based on previous work, we expect significant medium-large effects. Correlation between repeated observations within-subject will be modeled with structured variance-covariance and/or random subject effects. The best-fitting model will be chosen based on information criteria. Least-square (LS) means will be compared and plotted post-hoc to examine significant effects. Power Analysis: Using G*power, we estimate achieving 80% power to detect small effects (f=0.15) with 62 subjects in each of two groups, two timepoints, p=0.05, correlation among measures 0.3, and sphericity assumed.

3. Target engagement will be related to decrease in BPD symptom severity in the mbNF sample, (n=62)

We will use Pearson correlation to test the relationships between resting state DMN connectivity outcomes (pre- to post-NF change in within-DMN connectivity and DMN-FPCN anticorrelation) and BPD symptom severity (change in BSL23 score from pre-NF to primary endpoint). Power analysis: Using G*power,we estimate achieving 80% power to detect small effects (r = 0.21) with 124 subjects, alpha 0.05.

4. (exploratory): How long do neural and symptom changes last after mbNF + DBT?
We hypothesize that changes in neural connectivity and decreases in BPD symptom severity will last for several months after mbNF. We will explore the durability of observed changes using linear mixed-effects models testing for group (mbNF vs. controlNF) x timepoint (pre-NF to post- NF, 5 timepoints during DBTsg, and at 1- and 2-month post-DBT follow-up timepoints) in symptom change (primary: BSL23; secondary: SMS). Models will include a random effect for subject and fixed-effect covariates (e.g., age, sex, site, baseline symptoms).

Rigor and Reproducibility

The proposed study will take a number of steps to ensure reproducibility and rigor: (1) ensuring sufficient power;(2) administering reliable, valid, and developmentally appropriate assessments; (3) including age cutoffs to minimize variability of differences in brain function and disease phase due to late life changes in neural function; (4) creating a detailed statistical plan; (5) recruiting a representative sample, increasing the generalizability of findings; and (6) facilitating external reproducibility as de-identified data will be placed in the NDA

5.4.2 Analysis of Subject Characteristics

<u>*Sex as a Biological Variable*</u> The extent of sex differences in the neurobiology and behavioral expression of BPD remain unclear. [73] We will test sex differences in all variables as well as sex as a moderator of key predictors. Given well-established sex differences in the frequency of patients being given the BPD diagnosis and presentation for mental healthcare (despite likely-similar community prevalence),[73] we expect that our sample will be ~80% female. Although not our main aim, we will provide important preliminary results regarding sex differences in response to mbNF.

<u>*Site Differences*</u> Though we aim to minimize site differences in sociodemographic factors, we will probe potential differences and control for these in analyses. To further control for potential differences between the two sites, we will implement ComBat harmonization to remove site-related variance in connectivity measures. ComBat has been shown to facilitate reliable and efficient analysis, accounting for non-biological, scanner-specific sources of variability, in multi-site fMRI neuroimaging studies.[74] Additionally, all analyses will include a main effect of site, and we will examine potential first-order site x time and site x group x time interactions.

To further understand effects for the neural and clinical targets, we will control for key covariates (e.g., age, sex, site, head motion, clinical and demographic variables that differ between groups), and explore the impacts of mbNF vs. controlNF on the use of mindfulness skills and DBT skills from each module in daily life, DBTsg group attendance, and use of mental healthcare resources outside the study. We will also relate acceptability and tolerability data from program evaluation to attendance and dropout data.

As an exploratory analysis of broader modulation within the DMN, we will test within-DMN connectivity between other common nodes, i.e. posteromedial cortex, angular gyrus, lateral and medial temporal cortex. Furthermore, to explore the spatial specificity of our findings, we will perform this model on DMN connectivity voxel-wise (mPFC seed) across the brain using Threshold Free Cluster Enhancement (TFCE) for multiple comparisons correction.

5.4.3 Interim Analysis

This project is expected to be funded by the NIMH through the R61/R33 mechanism. This mechanism has a go/no-go decision after two years (the R61 phase).
For the GO criterion, we will test if mbNF leads to larger changes than control-NF (medium effect-size, Cohen's d of at least 0.5) in at least one of our two DMN connectivity targets. We will calculate within-subject change from pre- to post-NF, then use one-tailed t-tests (mbNF vs. shamNF) to test our hypothesis that mbNF will lead to larger change by decreasing within-DMN connectivity and/or increasing DMN-FPCN anticorrelation. This analysis will include all of the R61 participants who complete the neurofeedback day (n = 26 per group).

5.4.4 Handling of Missing Data

Missing data will be accommodated using multiple imputation or weighting procedures.

6 Trial Administration

6.1 Ethical Considerations: Informed Consent/Assent and HIPAA Authorization

Consent forms will be Institutional Review Board (IRB)-approved and the participant will be asked to read and review the document.

If a potential participant joins a first screening visit (always a live meeting, usually remote, with a Fineberg lab staff member), the first step will be the consent process, including a review of study overview and goals, study procedures, study risks and benefits, confidentiality, economic considerations, and in-person expectations regarding infection control for MRI days. Particular mention will be made and reviewed that participation is always voluntary, and that they may withdraw at any time without prejudice. Then the participant will have unlimited time to review the e-consent form, consult with trusted others in their life, think about it, and ask questions. Only if they decide to sign and submit the form does the visit proceed.

Upon submission, a copy of the informed consent document will be emailed to the participant/LAR for their records.

6.2 Institutional Review Board (IRB) Review

The protocol will be submitted to the IRB for review and approval. Approval of the protocol must be obtained before initiating any research activity. Any change to the protocol or study team will require an approved IRB amendment before implementation. The IRB will determine whether informed consent and HIPAA authorization are required.

A study closure report will be submitted to the IRB after all research activities have been completed.

6.3 Subject Confidentiality

Participant confidentiality and privacy is strictly held in trust by the participating investigators, their staff, and the sponsor(s) and their interventions. Therefore, the study protocol, documentation, data, and all other information generated will be held in strict confidence. No information concerning the study or the data will be released to any unauthorized third party without prior written approval of the sponsor.

All research activities will be conducted in as private a setting as possible.

The study monitor, representatives of the Institutional Review Board (IRB), or regulatory agencies may inspect all documents and records required to be maintained by the investigator, including but not limited to, medical records (office, clinic, or hospital) for the participants in this study. The clinical study sites will permit access to such records.

The study participant's contact information may be securely stored at each clinical site involved with the subject for internal use during the study. At the end of the study, all records will continue to be kept in a secure location for as long a period as dictated by the reviewing IRB, Institutional policies, or, if applicable, sponsor requirements.

Study participant data related to participation in the psychotherapy program will be maintained according to standard practice at the U Mass Psychological Services Center. These records will include participant identifiers and contact information.

Study participant research data, which is for purposes of statistical analysis and scientific reporting, will be transmitted to and stored at in Yale REDCap servers and on MR data servers at Yale and MGH. This will not include the participant's contact or identifying information. Rather, individual participants and their research data will be identified by a unique study identification number. The study data entry and study management systems used will be secured and password protected. At the end of the study, all study databases will be de-identified and archived electronically in Yale-maintained systems (for clinical, interview, and survey data) or Yale/MGH-maintained systems (for MR data).

6.4 Deviations/Unanticipated Problems

A protocol deviation is any noncompliance with the study protocol. The noncompliance may be either on the part of the participant, the investigator, or the study site staff. As a result of deviations, corrective actions are to be developed by the site and implemented promptly.

It is the responsibility of the site investigator to identify and report deviations within [specify number] working days of identification of the protocol deviation. All deviations must be addressed in study source documents, reported to the study sponsor, and the reviewing Institutional Review Board (IRB) per their policies.

Unanticipated problems involving risks to participants or others include, in general, any incident, experience, or outcome that meets all of the following criteria:

- Unexpected in terms of nature, severity, or frequency given (a) the research procedures that are described in the protocol-related documents, such as the Institutional Review Board (IRB)-approved research protocol and informed consent document; and (b) the characteristics of the participant population being studied;
- Related or possibly related to participation in the research (“possibly related” means there is a reasonable possibility that the incident, experience, or outcome may have been caused by the procedures involved in the research); and
- Suggests that the research places participants or others at a greater risk of harm (including physical, psychological, economic, or social harm) than was previously known or recognized.

The investigator will report unanticipated problems (UPs) to the reviewing Institutional Review Board (IRB) and to the study sponsor. The UP report will include the following information:

- Protocol identifying information: protocol title and number, PI's name, and the IRB project number;
- A detailed description of the event, incident, experience, or outcome;
- An explanation of the basis for determining that the event, incident, experience, or outcome represents an UP;
- A description of any changes to the protocol or other corrective actions that have been taken or are proposed in response to the UP.

To satisfy the requirement for prompt reporting, UPs will be reported using the following timeline:

- UPs that are serious adverse events (SAEs) will be reported to the IRB and study sponsor, if applicable within [insert timeline in accordance with of the investigator becoming aware of the event.
- Any other UP will be reported to the IRB and study sponsor within 5 days of the investigator becoming aware of the problem.
- All UPs should be reported to appropriate institutional officials (as required by an institution's written reporting procedures), the supporting agency head (or designee), and the Office for Human Research Protections (OHRP) within & [insert timeline in accordance with policy] of the IRB's receipt of the report of the problem from the investigator.
-

6.5 Data Safety Monitoring Plan

Summary of the protocol

This is a clinical trial where 124 adults with borderline personality disorder (52 in the R61 phase) will participate in an intervention that combines psychotherapy and mindfulness-focused real time fMRI neurofeedback (mbNF). Subjects will be randomized to receive either this mbNF targeting Default Mode Network connectivity (the experimental intervention) or yoked sham feedback (the control intervention). All subjects must be 18-60 years old, have BPD, no primary psychotic disorder, no bipolar disorder, no cognitive or learning disorder or serious head injury, no serious substance use disorder in last 6 months, be safe for and able to tolerate an MRI. Medications must be kept constant during the whole study. Subjects and clinical staff are blind regarding which group they are assigned to. We assess whether the experimental intervention yields greater improvements in emotion regulation and greater symptom improvements than the control intervention. We also assess changes in functional connectivity induced by the intervention and their relationships to changes in mindfulness and clinical symptoms.

Roles and Responsibilities: Dr. Fineberg, Dr. Hampson, and the Yale Human Research Protection Program (HRPP) will work together to monitor the safety of subjects at the Yale site. Yale will serve as the single IRB for the whole study. Dr. Whitfield-Gabrieli and MGH clinician Beth Brewer MSW will monitor the safety of subjects during in-person scan days at the MGH site, and remain in close contact with the whole leadership team about any concerns. Dr. Dixon-Gordon's team at U Mass Amherst will see the study subjects in remote DBT skills group each week and will raise any concerns with the rest of the study leadership team. In addition to day-to-day oversight, Dr. Fineberg will formally review all the clinical assessments on a quarterly basis to identify any potentially concerning patterns. The clinical raters and therapy teams will be blinded to subject group (mbNF or shamNF), but the neurofeedback staff (Dr. Whitfield-Gabrieli, Dr. Hampson and the at-scanner research assistants) will not be blinded, so they will review the study data quarterly as well to identify any potentially concerning patterns by group. A DSMB will be constituted by the study leadership team in consultation with the Program Officer in order to ensure that people with expertise in BPD and clinical trials are at the table. The study leadership team (Drs. Fineberg, and as needed, Drs. Hampson, Whitfield-Gabrieli, Dixon-Gordon, and Papa) will meet with the DSMB every six months to review all clinical data and discuss any potential clinical safety issues. The study staff that collect adverse events information will report serious and adverse events and UPIRSOs immediately to Dr. Fineberg who will review them to determine if further action is necessary. If there is any doubt, they will be discussed with the DSMB and IRB and officially reported and handled as recommended.

Any unanticipated or serious adverse events will be reported immediately to Dr. Fineberg who will report them to the NIH as required Unanticipated or serious events will also be discussed immediately by Dr. Fineberg and Dr. Dixon-Gordon, including the rest of the leadership team as needed, to assess their likelihood of being related to the intervention and to determine, in consultation with the IRB, if the study protocol should be modified or stopped.

Our team is experienced in the management of clinical risk in BPD in both clinical and clinical research settings. The clinical (Dr. Dixon-Gordon) and clinical assessment (Dr. Fineberg) leaders on this project will work together with the study team, a DSMB, and the Yale IRB to ensure careful monitoring and decision-making and to ensure that known risks (including self-harm, safety in the MR scanning environment, data privacy, and other clinical developments requiring a change of care), as well as any unexpected safety issues that may arise, are appropriately managed.

Trial Safety: The full study team will be responsible for maintaining confidentiality and security of all the data collected in the study, and for ensuring safety in the study. Dr. Fineberg will take primary responsibility for safety at the Yale site, and Dr. Whitfield-Gabrieli will take primary responsibility for safety at the MGH site. Dr. Fineberg will also take responsibility for the overall management of safety across all sites. The imaging centers at both Yale and MGH have established protocols for imaging safety that have been used effectively for many years. Drs. Hampson and Whitfield-Gabrieli will ensure that all study staff are well-trained in these safety protocols and committed to following them at their respective sites. In terms of privacy, all assessments will be conducted in private rooms, remote clinical sessions will be conducted over secure platforms, electronic data will be stored on firewall protected computers, and paper data will be stored in locked file cabinets in locked offices, and only accessible to approved study personnel. In terms of medication or treatment needs, if a situation arises in which a patient becomes clinically unstable and requires (or strongly desires) a change in medication or other treatment intervention that is a contraindication to ongoing study participation, they will pursue the required intervention through standard channels (i.e. their established providers), without any restrictions deriving from study participation. The decision to exclude them from the study will be made on a case-by-case basis by the study leadership team, blind to neurofeedback condition.

At each study assessment session, staff will collect adverse event info as well as clinically evaluating the patient. Any nontrivial clinical decline, or any serious adverse event, will be brought to the attention of the clinical team within 24 hours. Dr. Fineberg will be available throughout the study to field any questions or clinical concerns that may arise. These mechanisms – regular assessments, on-call availability, and direct coordination between the study clinical team (Drs. Fineberg and Dixon-Gordon; Beth Brewer) – will be used to manage any unexpected clinical situations that may arise, and to make decisions with the patient regarding any decision to withdraw from the protocol. If withdrawal from the study is necessary, we will provide appropriate referrals for other treatments. Incidental findings in the MR scanning session will be shared with a neuroradiologist at the relevant site, who will decide whether subjects should be notified and whether clinical follow-up is recommended.

Conflicts of interest (COI) are regularly monitored. The investigators do not currently have any COI of relevance to this study. However, if any unforeseen COI do develop, they will be reported to Yale and managed as recommended.

Reportable events: Suspension or termination of the protocol will be reported to NIH within 3 business days, along with an explanation. The death of a subject will be reported to NIH within 5 business days of the PI learning of it. Any unanticipated problems involving risks to subjects or others that are related or possibly related to participation will be reported within 7 days of the team becoming aware of the event. Any noncompliance the internal review board considers to be serious or continuing with be reported within 10 days to NIH. All adverse events and protocol violations in the study will be summarized in the yearly reports, including the renewal of the human subjects protocol of that site, and the annual progress

report to NIH. Documentation of reportable events will include identifying information for the research protocol, date of event, date when PI became aware of event, detailed description and impact of event on subject (or other), summary of any measures taken and required reports made to regulatory bodies, as well as any changes in the protocol or other corrective actions taken.

Data management, analysis and quality assurance: Data collected will include personal health information, clinical assessments and MR imaging data. All assessments will be conducted in private rooms, electronic data will be stored on firewall protected computers, and paper data will be stored in locked file cabinets in locked offices, and only accessible to approved study personnel. All personal will be trained to follow HIPAA regulations. The full study team will be responsible for overseeing quality assurance in the data, which will involve cross-checking data records with original sources and verifying accurate data analyses.

Risks associated with the current study are deemed greater than minimal for the following reasons:

The proposed study involves greater than minimal risk because the subject population (adults with Borderline Personality Disorder) has a higher than average frequency of self-harm and because this study involves a treatment intervention. Given the established safety of both fMRI neurofeedback and DBT skills group, we do not view the proposed study as high risk.

Although we have assessed the proposed study as one of greater than minimal risk, the potential exists for anticipated and/or unanticipated adverse events, serious or otherwise, to occur since it is not possible to predict with certainty the absolute risk in any given individual or in advance of first-hand experience with the proposed study methods. Therefore, we provide a plan for monitoring the data and safety of the proposed study as follows:

Attribution of Adverse Events

Adverse events will be monitored for each subject participating in the study and attributed to the study procedures/design by the principal investigators. Dr. Fineberg will have primary responsibility for any adverse events of clinical significance or that involves overall patient well-being. Dr. Hampson will be responsible for any adverse events that occur during MR scanning at Yale, and Dr. Whitfield-Gabrieli for any adverse events that occur during MR

scanning at MGH. These events will be attributed to the study procedures/design according to the following categories:

a.) Definite: Adverse event is clearly related to investigational procedures(s).

b.) Probable: Adverse event is likely related to investigational procedures(s).

c.) Possible: Adverse event may be related to investigational procedures(s).

d.) Unlikely: Adverse event is likely not to be related to the investigational procedures(s).

e.) Unrelated: Adverse event is clearly not related to investigational procedures(s).

Plan for Grading Adverse Events:

The following scale will be used in grading the severity of adverse events noted during the study:

Mild adverse event

Moderate adverse event

Severe adverse event

Plan for Determining Seriousness of Adverse Events:

Serious Adverse Events:

In addition to grading the adverse event, the PIs will determine whether the adverse event meets the criteria for a Serious Adverse Event (SAE). An adverse event is considered serious if it results in any of the following outcomes:

Death;

A life-threatening experience in-patient hospitalization or prolongation of existing hospitalization;

A persistent or significant disability or incapacity;

A congenital anomaly or birth defect; OR

Any other adverse event that, based upon appropriate medical judgment, may jeopardize the subject’s health and may require medical or surgical intervention to prevent one of the other outcomes listed in this definition.

An adverse event may be graded as severe but still not meet the criteria for a Serious Adverse Event. Similarly, an adverse event may be graded as moderate but still meets the criteria for an SAE. It is important for the PIs to consider the grade of the event as well as its "seriousness" when determining whether reporting to the IRB is necessary.

Plan for reporting UPIRSOs (including Adverse Events) to the IRB

The principal investigators will report the following types of events to the IRB:

Any incident, experience or outcome that meets ALL 3 of the following criteria:

Is unexpected (in terms of nature, specificity, severity, or frequency) given (a) the research procedures described in the protocol-related documents, such as the IRB-approved protocol and informed consent document and (b) the characteristics of the subject population being studied; AND

Is related or possibly related to participation in the research (possibly related means there is a reasonable possibility that the incident, experience, or outcome may have been caused by the procedures involved in the research); AND

Suggests that the research places subjects or others at greater risk of harm (including physical, psychological, economic, legal, or social harm) than was previously known or recognized.

Unanticipated Problems Involving Risks to Subjects or Others (UPIRSOs) may be medical or non-medical in nature and include – but are not limited to – serious, unexpected, and related adverse events and unanticipated adverse device effects.

These UPIRSOs/SAEs will be reported to the IRB in accordance with IRB Policy 710, using the appropriate forms found on the website. All related events involving risk but not meeting the prompt reporting requirements described in IRB Policy 710 will be reported to the IRB in summary form at the time of continuing review. If appropriate, such summary may be a simple brief statement that events have occurred at the expected frequency and level of severity as previously documented. In lieu of a summary of external events, we may submit a current DSMB report.

Adverse events will be reported to Yale University in a timely manner, according to the IRB guidelines. Any serious and possibly, probably, or definitely related events will be reported

within 5 days of discovery to both the Yale IRB and the DSMB. All adverse events (whether possibly related or not) will be summarized for NIH in the yearly renewal report.

Plan for reporting adverse events to co-investigators on the study, Protocol Review Committee (PRC), DSMB, , funding agency, and regulatory and decision-making bodies.

For the current study, the following individuals, funding, and/or regulatory agencies will be notified:

All Co-Investigators listed on the protocol.

National Institutes of Health

DSMB

Every 6 months, the study PIs and co-Is will conduct a review of all adverse events upon the completion of every study subject. The principal investigator will evaluate the frequency and severity of the adverse events and determine if modifications to the protocol or consent form are required.

No injuries are anticipated from participation in this study. No special provisions for medical treatment are included with this study. Participants will be able to access their usual providers or the emergency department should they require evaluation or treatment during the study.

6.6 Data Quality Assurance

Study staff will be trained to good inter-rater reliability for study assessments, and to adherence for mindfulness training and DBT skills group psychotherapy.

6.7 Study Records

Regulatory documents

Protocol

Consent forms

Interview results

Surveys

Study visit notes

Psychotherapy records

MRI data

6.8 Access to Source

Source data will be maintained per Medical Records policy in a password protected, secure, Health Insurance Portability and Accountability Act (HIPAA) compliant, web-based electronic database with a built-in audit trail.
Only Institutional Review Board (IRB) approved research team members who have current HIPAA and Collaborative Institutional Training Initiative (CITI) Good Clinical Practice (GCP) and human subjects protection training will be authorized to access records.

6.9 Data or Specimen Storage/Security

Data will be stored using secure electronic storage systems, including

6.10 Retention of Records

We will store de-identified records indefinitely. We will destroy identifiers within one year after planned analyses are complete.

6.11 Study Modification

Study modification requests will be submitted to IRB prior to any changes being made to study procedures.

6.12 Study Completion

The study is complete when 124 participants have completed all study activities. We will notify the IRB after the last participant completes the last study procedure.

6.13 Funding Source

We anticipate funding from the National Institutes of Mental Health

6.14 Publication Plan

We expect to publish the results of this study in peer-reviewed scientific journals, using the data in ways that protect confidentiality, by publishing group-level data, de-identified numerical results, and possibly de-identified quotes that represent the sentiments of a group, taking extreme care to never identify an individual in the presentation of results.

7 References

1. Lenzenweger, M.F., et al., *DSM-IV personality disorders in the National Comorbidity Survey Replication.* Biol Psychiatry, 2007. **62**(6): p. 553-64.
2. Zanarini, M.C., et al., *Attainment and stability of sustained symptomatic remission and recovery among patients with borderline personality disorder and axis II comparison subjects: a 16-year prospective follow-up study.* Am J Psychiatry, 2012. **169**(5): p. 476-83.
3. Cailhol, L., et al., *Prevalence, Mortality, and Health Care Use among Patients with Cluster B Personality Disorders Clinically Diagnosed in Quebec: A Provincial Cohort Study, 2001-2012.* Can J Psychiatry, 2017. **62**(5): p. 336-342.
4. Wedig, et al., *Predictors of suicide threats in patients with borderline personality disorder over 16 years of prospective follow-up.* Psychiatry Res, 2013. **208**(3): p. 252-6.
5. Paris, J. and H. Zweig-Frank, *A 27-year follow-up of patients with borderline personality disorder.* Compr Psychiatry, 2001. **42**(6): p. 482-7.
6. Boisseau, C.L., et al., *Individuals with single versus multiple suicide attempts over 10years of prospective follow-up.* Compr Psychiatry, 2013. **54**(3): p. 238-42.
7. Pompili, M., et al., *Suicide in borderline personality disorder: a meta-analysis.* Nord J Psychiatry, 2005. **59**(5): p. 319-24.
8. (NICE), N.I.f.H.a.C.E., *Borderline Personality Disorder: recognition and management Clinical Guideline CG78*. 2009.
9. *Clinical Practice Guideline for the Management of Borderline Personality Disorder*, N.H.a.M.R. Council, Editor. 2012, National Health and Medical Research Council: Melbourne.
10. Storebø, O.J., et al., *Psychological therapies for people with borderline personality disorder.* Cochrane Database Syst Rev, 2020. **5**(5): p. Cd012955.
11. Cristea, I.A., et al., *Efficacy of Psychotherapies for Borderline Personality Disorder: A Systematic Review and Meta-analysis.* JAMA Psychiatry, 2017. **74**(4): p. 319-328.
12. Oud, M., et al., *Specialized psychotherapies for adults with borderline personality disorder: A systematic review and meta-analysis.* Aust N Z J Psychiatry, 2018. **52**(10): p. 949-961.
13. Barnicot, K. and M. Crawford, *Dialectical behaviour therapy v. mentalisation-based therapy for borderline personality disorder.* Psychol Med, 2019. **49**(12): p. 2060-2068.
14. Linehan, M.M., et al., *Dialectical behavior therapy for high suicide risk in individuals with borderline personality disorder: a randomized clinical trial and component analysis.* JAMA Psychiatry, 2015. **72**(5): p. 475-82.
15. Soler, J., et al., *Dialectical behaviour therapy skills training compared to standard group therapy in borderline personality disorder: a 3-month randomised controlled clinical trial.* Behav Res Ther, 2009. **47**(5): p. 353-8.

16. McMain, S.F., et al., *A randomized trial of brief dialectical behaviour therapy skills training in suicidal patients suffering from borderline disorder.* Acta Psychiatr Scand, 2017. **135**(2): p. 138-148.

17. Iliakis, E.A., G.S. Ilagan, and L.W. Choi-Kain, *Dropout rates from psychotherapy trials for borderline personality disorder: A meta-analysis.* Personal Disord, 2021. **12**(3): p. 193-206.

18. Kröger, C., et al., *Effectiveness, response, and dropout of dialectical behavior therapy for borderline personality disorder in an inpatient setting.* Behav Res Ther, 2013. **51**(8): p. 411-6.

19. Bateman, A.W., J. Gunderson, and R. Mulder, *Treatment of personality disorder.* Lancet, 2015. **385**(9969): p. 735-43.

20. Jerschke, S., et al., *[The treatment history of patients with borderline personality disorder in the Republic of Germany].* Fortschr Neurol Psychiatr, 1998. **66**(12): p. 545-52.

21. Shibata, K., et al., *Perceptual learning incepted by decoded fMRI neurofeedback without stimulus presentation.* Science, 2011. **334**(6061): p. 1413-5.

22. Shibata, K., et al., *Differential Activation Patterns in the Same Brain Region Led to Opposite Emotional States.* PLoS Biol, 2016. **14**(9): p. e1002546.

23. Amano, K., et al., *Learning to Associate Orientation with Color in Early Visual Areas by Associative Decoded fMRI Neurofeedback.* Curr Biol, 2016. **26**(14): p. 1861-6.

24. Taschereau-Dumouchel, V., et al., *Towards an unconscious neural reinforcement intervention for common fears.* Proc Natl Acad Sci U S A, 2018. **115**(13): p. 3470-3475.

25. Cortese, A., et al., *Multivoxel neurofeedback selectively modulates confidence without changing perceptual performance.* Nat Commun, 2016. **7**: p. 13669.

26. Zhang, J., et al., *Targeting default mode network connectivity with mindfulness-based fMRI neurofeedback: A pilot study among adolescents with affective disorder history*, in *Biorxiv*. 2022.

27. Young, K.D., et al., *Real-Time Functional Magnetic Resonance Imaging Amygdala Neurofeedback Changes Positive Information Processing in Major Depressive Disorder.* Biol Psychiatry, 2017. **82**(8): p. 578-586.

28. Bauer, C.C.C., et al., *Real-time fMRI neurofeedback reduces auditory hallucinations and modulates resting state connectivity of involved brain regions: Part 2: Default mode network -preliminary evidence.* Psychiatry Res, 2020. **284**: p. 112770.

29. Scheinost, D., et al., *Orbitofrontal cortex neurofeedback produces lasting changes in contamination anxiety and resting-state connectivity.* Translational psychiatry, 2013. **3**: p. e250.

30. Subramanian, L., et al., *Functional Magnetic Resonance Imaging Neurofeedback-guided Motor Imagery Training and Motor Training for Parkinson's Disease: Randomized Trial.* Front Behav Neurosci, 2016. **10**: p. 111.

31. Sukhodolsky, D.G., et al., *Randomized, Sham-Controlled Trial of Real-Time Functional Magnetic Resonance Imaging Neurofeedback for Tics in Adolescents With Tourette Syndrome.* Biol Psychiatry, 2020. **87**(12): p. 1063-1070.

32. Wupperman, P., C.S. Neumann, and S.R. Axelrod, *Do deficits in mindfulness underlie borderline personality features and core difficulties?* J Pers Disord, 2008. **22**(5): p. 466-82.

33. Roberts, A., et al., *Does trait mindfulness mediate the relationship between borderline personality symptoms and emotion dysregulation?* Borderline Personality Disorder and Emotion Dysregulation, 2023. **10**(1): p. 19.

34. Wupperman, P., et al., *The role of mindfulness in borderline personality disorder features.* J Nerv Ment Dis, 2009. **197**(10): p. 766-71.

35. Salgó, E., et al., *Emotion regulation, mindfulness, and self-compassion among patients with borderline personality disorder, compared to healthy control subjects.* Plos one, 2021. **16**(3): p. e0248409.

36. Kounidas, G. and S. Kastora, *Mindfulness training for borderline personality disorder: A systematic review of contemporary literature.* Personal Ment Health, 2022. **16**(3): p. 180-189.

37. Linehan, M., *Cognitive-behavioral treatment of borderline personality disorder*. Diagnosis and treatment of mental disorders. 1993, New York: Guilford Press. xvii, 558 p.

38. Carmona i Farres, C., et al., *Effects of mindfulness training on borderline personality disorder: Impulsivity versus emotional dysregulation.* Mindfulness, 2019. **10**(7): p. 1243-1254.

39. Elices, M., et al., *Impact of Mindfulness Training on Borderline Personality Disorder: A Randomized Trial.* Mindfulness, 2016. **7**: p. 584-595.

40. Feliu-Soler, A., et al., *Effects of dialectical behaviour therapy-mindfulness training on emotional reactivity in borderline personality disorder: preliminary results.* Clin Psychol Psychother, 2014. **21**(4): p. 363-70.

41. Mochrie, K.D., et al., *From the hospital to the clinic: The impact of mindfulness on symptom reduction in a DBT partial hospital program.* Journal of Clinical Psychology, 2019. **75**(7): p. 1169-1178.

42. Zeifman, R.J., et al., *The independent roles of mindfulness and distress tolerance in treatment outcomes in dialectical behavior therapy skills training.* Personal Disord, 2020. **11**(3): p. 181-190.

43. Sezer, I., D.A. Pizzagalli, and M.D. Sacchet, *Resting-state fMRI functional connectivity and mindfulness in clinical and non-clinical contexts: A review and synthesis.* Neurosci Biobehav Rev, 2022. **135**: p. 104583.

44. Visintin, E., et al., *Mapping the brain correlates of borderline personality disorder: A functional neuroimaging meta-analysis of resting state studies.* J Affect Disord, 2016. **204**: p. 262-9.

45. Schulze, L., C. Schmahl, and I.J.B.p. Niedtfeld, *Neural correlates of disturbed emotion processing in borderline personality disorder: a multimodal meta-analysis.* 2016. **79**(2): p. 97-106.

46. Schulze, L., C. Schmahl, and I. Niedtfeld, *Neural correlates of disturbed emotion processing in borderline personality disorder: a multimodal meta-analysis.* Biol Psychiatry, 2016. **79**(2): p. 97-106.

47. Iskric, A. and E. Barkley-Levenson, *Neural Changes in Borderline Personality Disorder After Dialectical Behavior Therapy-A Review.* Front Psychiatry, 2021. **12**: p. 772081.

48. Bauer, C.C.C., et al., *Mindfulness training reduces stress and amygdala reactivity to fearful faces in middle-school children.* Behav Neurosci, 2019. **133**(6): p. 569-585.

49. Lieberman, J.M., et al., *Posterior cingulate cortex targeted real-time fMRI neurofeedback recalibrates functional connectivity with the amygdala, posterior insula, and default-mode network in PTSD.* Brain Behav, 2023. **13**(3): p. e2883.

50. Wang, D., et al., *Parcellating cortical functional networks in individuals.* Nat Neurosci, 2015. **18**(12): p. 1853-60.

51. Bauer, R., et al., *Closed-loop adaptation of neurofeedback based on mental effort facilitates reinforcement learning of brain self-regulation.* Clinical Neurophysiology, 2016. **127**(9): p. 3156-3164.

52. Paret, C., et al., *Associations of emotional arousal, dissociation and symptom severity with operant conditioning in borderline personality disorder.* Psychiatry Res, 2016. **244**: p. 194-201.

53. Zaehringer, J., et al., *Improved emotion regulation after neurofeedback: A single-arm trial in patients with borderline personality disorder.* Neuroimage Clin, 2019. **24**: p. 102032.

54. Lakeman, R., et al., *Towards online delivery of Dialectical Behaviour Therapy: A scoping review.* Int J Ment Health Nurs, 2022. **31**(4): p. 843-856.

55. Dunn, N., et al., *Acceptability of Telehealth for Multidiagnostic Suicidal Patients in a Real-World Dialectical Behavior Therapy Clinic During the COVID-19 Pandemic.* Telemed J E Health, 2023. **29**(4): p. 593-601.

56. Hood, P., et al., *Making Lemonade out of Lemons: Dialectical Behavior Therapy via Telehealth During a Pandemic.* Behav Ther, 2023. **54**(5): p. 876-891.

57. Zalewski, M., et al., *Lessons Learned Conducting Dialectical Behavior Therapy via Telehealth in the Age of COVID-19.* Cogn Behav Pract, 2021. **28**(4): p. 573-587.

58. Hyland, K.A., et al., *Telehealth for dialectical behavioral therapy: A commentary on the experience of a rapid transition to virtual delivery of DBT.* Cognitive and Behavioral Practice, 2022. **29**(2): p. 367-380.

59. Bohus, M., et al., *The short version of the Borderline Symptom List (BSL-23): development and initial data on psychometric properties.* Psychopathology, 2009. **42**(1): p. 32-9.

60. Fox, M.D., et al., *The human brain is intrinsically organized into dynamic, anticorrelated functional networks.* Proc Natl Acad Sci U S A, 2005. **102**(27): p. 9673-8.

61. Tanay, G. and A. Bernstein, *State Mindfulness Scale (SMS): development and initial validation.* Psychol Assess, 2013. **25**(4): p. 1286-99.

62. Zanarini, M.C., Frankenburg F.R., Sickel, A.E., Yong, L., *The Diagnostic Interview for DSM-IV Personality Disorders.* , L.f.t.S.o.A.D. Mclean Hospital, Editor. 1996: Belmont, Mass.

63. First, M.B., Williams, J.B.W., Karg, R.S., Spitzer, R.L., *Structured Clinical Interview for DSM-5 - Research Version*. 2015, Arlington, VA: American Psychiatric Association.

64. Hinds, O., et al., *Computing moment-to-moment BOLD activation for real-time neurofeedback.* Neuroimage, 2011. **54**(1): p. 361-8.

65. Tisdall, M.D., et al., *Volumetric navigators for prospective motion correction and selective reacquisition in neuroanatomical MRI.* Magn Reson Med, 2012. **68**(2): p. 389-99.

APPROVED BY THE YALE UNIVERSITY IRB 7/28/2025

66. Dosenbach, N.U.F., et al., *Real-time motion analytics during brain MRI improve data quality and reduce costs.* Neuroimage, 2017. **161**: p. 80-93.

67. Sayadaw, C., *Practical Insight Meditation*.

68. Butler, D.J., et al., *A review of the benefits and limitations of a primary care-embedded psychiatric consultation service in a medically underserved setting.* Int J Psychiatry Med, 2018. **53**(5-6): p. 415-426.

69. Safran, J.D., *Towards a refinement of cognitive therapy in light of interpersonal theory: I. Theory.* Clinical Psychology Review, 1990. **10**(1): p. 87-105.

70. Okano, K., et al., *Real-time fMRI feedback impacts brain activation, results in auditory hallucinations reduction: Part 1: Superior temporal gyrus -Preliminary evidence.* Psychiatry Res, 2020. **286**: p. 112862.

71. Sinha, R. and K. Tuit, *Imagery scripts developmental procedures manual*. 2012, New Haven: Yale University School of Medicine.

72. McAuley, E., T. Duncan, and V.V. Tammen, *Psychometric Properties of the Intrinsic Motivation Inventory in a Competitive Sport Setting: A Confirmatory Factor Analysis.* Research Quarterly for Exercise and Sport, 1988. **60**(1): p. 48-58.

73. Qian, X., et al., *Sex differences in borderline personality disorder: A scoping review.* PLoS One, 2022. **17**(12): p. e0279015.

74. Yu, M., et al., *Statistical harmonization corrects site effects in functional connectivity measurements from multi-site fMRI data.* Hum Brain Mapp, 2018. **39**(11): p. 4213-4227.

APPROVED BY THE YALE UNIVERSITY IRB 7/28/2025
