# Supplementary material for: A protocol for MIndfulness-based Neurofeedback to augment DBT psychotherapy for adults with Borderline Personality Disorder (MIND-BPD)
Source: PLoS One. 2026 Mar 20;21(3):e0338002. doi: 10.1371/journal.pone.0338002 (PMC13004318; doi:10.1371/journal.pone.0338002)
Supplement: S2 File — (DOCX) [file pone.0338002.s002.docx]

**SPIRIT 2025 checklist of items to address in a randomized trial protocol***

| **Section / Topic** | **No** | **SPIRIT 2025 checklist item description** | **Reported on page no.** |
| --- | --- | --- | --- |
| **Administrative information** | | |  |
| Title and structured summary | 1a | Title stating the trial design, population, and interventions, with identification as a protocol  *A protocol for* ***MI****ndfulness-based* ***N****eurofeedback to augment* ***D****BT psychotherapy for adults with* ***B****orderline* ***P****ersonality* ***D****isorder (MIND-BPD)* | Title page (page 1) |
|  | 1b | Structured summary of trial design and methods, including items from the World Health Organization Trial Registration Data Set  *Information available in trial registration on ClinicalTrials.gov (****NCT06446765****). Link: https://clinicaltrials.gov/study/NCT06446765?term=yale%20fineberg&rank=2* | Abstract (page 3) |
| Protocol version | 2 | Version date and identifier  Version #8, July 23, 2025 | n/a (protocol provided as attachment) |
| Roles and responsibilities | 3a | Names, affiliations, and roles of protocol contributors | Title page (page 1), and Author Contributions (pages 27 -29) |
|  | 3b | Name and contact information for the trial sponsor  *This is an investigator-initiated trial. The investigators are listed as authors on the paper and funding acquisition is listed as a role in the Author Contributions.* | Title page (page 1), and Author Contributions (pages 27-29) |
|  | 3c | Role of trial sponsor and funders in design, conduct, analysis, and reporting of trial; including any authority over these activities  *This is an investigator-initiated trial (so the lead investigator is the sponsor). The funding comes from the National Institutes of Health (NIMH): they contributed to some final decisions about risk management practices. Day-to-day trial execution, analyses, and interpretation/reporting of data will be solely decisions of the study team.* | Declarations, Funding (page 27) |
|  | 3d | Composition, roles, and responsibilities of the coordinating site, steering committee, endpoint adjudication committee, data management team, and other individuals or groups overseeing the trial, if applicable  *N/A* | N/A |
| **Open science** | | |  |
| Trial registration | 4 | Name of trial registry, identifying number (with URL), and date of registration. If not yet registered, name of intended registry  *Please find trial registration on ClinicalTrials.gov (****NCT06446765****). Link:* [*https://clinicaltrials.gov/study/NCT06446765?term=yale%20fineberg&rank=2*](https://clinicaltrials.gov/study/NCT06446765?term=yale%20fineberg&rank=2)*.*  *Registered March 13, 2025, last update June 5, 2025.* | Abstract (page 3), Methods (page 7) |
| Protocol and statistical analysis plan | 5 | Where the trial protocol and statistical analysis plan can be accessed  *Trial protocol with statistical analysis plan is included with the submission.* |  |
| Data sharing | 6 | Where and how the individual de-identified participant data (including data dictionary), statistical code, and any other materials will be accessible  *De-identified data will be made publicly available via the National Institute of Mental Health Data Archive.* | See section “Data management, sharing, and monitoring”, page 25 |
| Funding and conflicts of interest | 7a | Sources of funding and other support (e.g., supply of drugs)  *This project is supported by NIH (R61MH135009). SWG is supported in part by the generous support of the Tommy Fuss Endowed Chair in Precision Psychiatry at Massachusetts General Hospital. The work described in this article was funded in part by the State of Connecticut, Department of Mental Health and Addiction Services, but this publication does not express the views of the Department of Mental Health and Addiction Services or the State of Connecticut. The views and opinions expressed are those of the authors.* | See section “Funding”, page 27 |
|  | 7b | Financial and other conflicts of interest for principal investigators and steering committee members  *SKF and JP have done ad hoc consulting to Boehringer Ingelheim GmBH. SKF also has consulted for atai Life Sciences and serves on a Scientific Advisory Board for Oryzon. KDG receives payment for training and consultation in DBT, and royalties from American Psychological Association for publications. The other authors have no relationships to disclose.* | See section “Disclosures”, page 27 |
| Dissemination policy | 8 | Plans to communicate trial results to participants, healthcare professionals, the public, and other relevant groups (e.g., reporting in trial registry, plain language summary, publication)  *Data will be analyzed during ongoing acquisition to ensure quality and to present for scientific audiences at conferences. Once data collection is complete, final analyses will be posted as pre-prints and submitted to peer-reviewed journals and scientific conferences. We will also seek out opportunities to give oral presentations and webinars to patient and family stakeholder communities.* |  |
| **Introduction** | | |  |
| Background and rationale | 9a | Scientific background and rationale, including summary of relevant studies (published and unpublished) examining benefits and harms for each intervention | See section “Introduction”, pages 4-6 |
|  | 9b | Explanation for choice of comparator  *In the active mbNF condition, neurofeedback will be derived from DMN-CEN activity. In the sham condition, feedback will be derived from another participant’s active timeseries (a yoked-sham control).* | See section “Neurofeedback: control condition”, pages 15 |
| Objectives | 10 | Specific objectives related to benefits and harms |  |
| **Methods: Patient and public involvement, trial design** | | |  |
| Patient and public involvement | 11 | Details of, or plans for, patient or public involvement in the design, conduct, and reporting of the trial  *This study team includes an investigator with lived-experience of BPD who serves as a consultant to the overall project and who leads the program evaluation component of the study. Ms. Papa has been involved in the project since grant writing and will continue to be involved through reporting and will support efforts to disseminate this work to the patient and family stakeholder communities.* | See section “Lived experience-led program evaluation”, page 20-21 |
| Trial design | 12 | Description of trial design including type of trial (e.g., parallel group, crossover), allocation ratio, and framework (e.g., superiority, equivalence, non-inferiority, exploratory)  *We will conduct a randomized controlled superiority trial enrolling 52 adults with Borderline Personality Disorder aged 18-60 years. Participants will be randomized to either an mbNF (N=26) or sham-NF (N=26) condition, and network connectivity will be measured pre- and post-intervention.* | See section “Methods: Study Overview”, page 6-7 |
| **Methods: Participants, interventions, and outcomes** | | |  |
| Trial setting | 13 | Settings (e.g., community, hospital) and locations (e.g., countries, sites) where the trial will be conducted  *Study participants (52 adults with BPD), aged 18-60 years old, will be recruited from the New Haven and Boston areas by staff at the Fineberg lab housed at the Yale School of Medicine and Connecticut Mental Health Center (New Haven, CT).*  *Participants will attend remote video-conferenced clinical meetings for baseline characterization, imaging sessions at Yale Magnetic Resonance Research Center (MRRC) or Athinoula A. Martinos Center for Biomedical Imaging (Boston, MA).* | See sections: Methods: Recruitment (page 7), Study Interventions: Mindfulness-based Neurofeedback (mbNF) (page 11) |
| Eligibility criteria | 14a | Eligibility criteria for participants  *Eligibility criteria are summarized in the text and in Table 1.* | See page 8, Table 1 |
|  | 14b | If applicable, eligibility criteria for sites and for individuals who will deliver the interventions (e.g., surgeons, physiotherapists) | N/A |
| Intervention and comparator | 15a | Intervention and comparator with sufficient details to allow replication including how, when, and by whom they will be administered. If relevant, where additional materials describing the intervention and comparator (e.g., intervention manual) can be accessed | See study interventions: randomization and blinding (page 11), “Neurofeedback: control condition”, pages 15 |
|  | 15b | Criteria for discontinuing or modifying allocated intervention/comparator for a trial participant (e.g., drug dose change in response to harms, participant request, or improving/worsening disease)  *The participant experience and level of risk are the same in both conditions, and the intervention (real or sham) will be stopped immediately if a participant feels uncomfortable during the scan.* | See study interventions: randomization and blinding (page 11) |
|  | 15c | Strategies to improve adherence to intervention/comparator protocols, if applicable, and any procedures for monitoring adherence (e.g., drug tablet return, sessions attended)  *Adherence to the rtNF protocol will be assessed with a self report measure as part of the post-NF program evaluation survey and the Intrinsic Motivation Inventory: at the end of each neurofeedback run, participants will indicate how often they were using the mindfulness practice.* | See section “Lived experience-led program evaluation”, page 20-21, Figure 1 |
|  | 15d | Concomitant care that is permitted or prohibited during the trial  *Participants are asked to maintain steady psychotherapy and medications during the trial. People who are enrolled in formal DBT psychotherapy are not eligible. People who are taking sedating medications are not eligible.* | See page 8, Table 1. |
| Outcomes | 16 | Primary and secondary outcomes, including the specific measurement variable (e.g., systolic blood pressure), analysis metric (e.g., change from baseline, final value, time to event), method of aggregation (e.g., median, proportion), and time point for each outcome  *See statistical design and power analysis section* | Page 23-24 |
| Harms | 17 | How harms are defined and will be assessed (e.g., systematically, non-systematically)  *See risk management section, lived experience program evaluation.* | Page 20-21, 21-23 |
| Participant timeline | 18 | Time schedule of enrollment, interventions (including any run-ins and washouts), assessments, and visits for participants. A schematic diagram is highly recommended (see Figure)  *See figure 1, Study Design* | Figure 1, page 9-10 |
| Sample size | 19 | How sample size was determined, including all assumptions supporting the sample size calculation  *See power analysis in Statistical Design and Power Analysis* | Page 23-24 |
| Recruitment | 20 | Strategies for achieving adequate participant enrollment to reach target sample size  *Participants are recruited through well-established methods using social media advertisement, this is described in methods: recruitment section.* | Page 7 |
| **Methods: Assignment of interventions** | | |  |
| Randomization: |  |  |  |
| Sequence generation | 21a | Who will generate the random allocation sequence and the method used  *The randomization sequence is generated by the neuroimaging staff.* | Page 10-11 |
|  | 21b | Type of randomization (simple or restricted) and details of any factors for stratification. To reduce predictability of a random sequence, other details of any planned restriction (e.g., blocking) should be provided in a separate document that is unavailable to those who enroll participants or assign interventions | Page 10-11 |
| Allocation concealment  mechanism | 22 | Mechanism used to implement the random allocation sequence (e.g., central computer/telephone; sequentially numbered, opaque, sealed containers), describing any steps to conceal the sequence until interventions are assigned | Page 10-11 |
| Implementation | 23 | Whether the personnel who will enroll and those who will assign participants to the interventions will have access to the random allocation sequence | Page 10-11 |
| Blinding | 24a | Who will be blinded after assignment to interventions (e.g., participants, care providers, outcome assessors, data analysts) | Page 10-11 |
|  | 24b | If blinded, how blinding will be achieved and description of the similarity of interventions | Page 10-11 |
|  | 24c | If blinded, circumstances under which unblinding is permissible, and procedure for revealing a participant’s allocated intervention during the trial  *Participants’ group assignment will be revealed to them at the end of the trial.* | Page 11 |
| **Methods: Data collection, management, and analysis** | | |  |
| Data collection methods | 25a | Plans for assessment and collection of trial data, including any related processes to promote data quality (e.g., duplicate measurements, training of assessors) and a description of trial instruments (e.g., questionnaires, laboratory tests) along with their reliability and validity, if known. Reference to where data collection forms can be accessed, if not in the protocol | Figure 1, Methods page 6-16 |
|  | 25b | Plans to promote participant retention and complete follow-up, including list of any outcome data to be collected for participants who discontinue or deviate from intervention protocols  *All outcomes in the R61 study are collected in the same study session as randomization.* | N/A |
| Data management | 26 | Plans for data entry, coding, security, and storage, including any related processes to promote data quality (e.g., double data entry; range checks for data values). Reference to where details of data management procedures can be accessed, if not in the protocol  *Data are entered directly into the electronic database that is a HIPAA-compliant instance of REDCap maintained by Yale University School of Medicine* | Page 25 |
| Statistical methods | 27a | Statistical methods used to compare groups for primary and secondary outcomes, including harms | Page 23-24 |
|  | 27b | Definition of who will be included in each analysis (e.g., all randomized participants), and in which group | Page 23-24 |
|  | 27c | How missing data will be handled in the analysis | Page 23-24 |
|  | 27d | Methods for any additional analyses (e.g., subgroup and sensitivity analyses) | Page 23-24 |
| **Methods: Monitoring** | | |  |
| Data monitoring committee | 28a | Composition of data monitoring committee (DMC); summary of its role and reporting structure; statement of whether it is independent from the sponsor and funder; conflicts of interest and reference to where further details about its charter can be found, if not in the protocol. Alternatively, an explanation of why a DMC is not needed  *An independent Data and Safety Monitoring Board (DSMB) has been established for this study, composed of three voting members: a biostatistician, a neuropsychologist specialized in BPD, a psychiatrist specializing in BPD. The DSMB is fully independent from the sponsor and funder, and all members attest to the absence of conflicts of interest. The board meets every six months to review trial conduct, including participant safety, adverse events, and data quality. The DSMB chair oversees meetings, sets the agenda, and serves as the point of contact. Further details on DSMB procedures, including its charter, are available upon request from the lead investigators.* | Page 7 |
|  | 28b | Explanation of any interim analyses and stopping guidelines, including who will have access to these interim results and make the final decision to terminate the trial | N/A |
| Trial monitoring | 29 | Frequency and procedures for monitoring trial conduct. If there is no monitoring, give explanation  *In addition to DSMB oversight, the study team will meet weekly to monitor trial conduct, coordinate across local sites, and ensure timely resolution of any operational issues.* | Study Overview, page 6-7, Program evaluation page 20-21 |
| **Ethics** | | |  |
| Research ethics approval | 30 | Plans for seeking research ethics committee/institutional review board approval  *This study has been approved by the Yale University Institutional Review Board #2000037769* | Recruitment, page 7 |
| Protocol amendments | 31 | Plans for communicating important protocol modifications to relevant parties  *All IRB modifications will be disseminated to the investigator teams.* | Study Overview, page 6-7. |
| Consent or assent | 32a | Who will obtain informed consent or assent from potential trial participants or authorized proxies, and how  *Consent process* | Page 7-8 |
|  | 32b | Additional consent provisions for collection and use of participant data and biological specimens in ancillary studies, if applicable | N/A |
| Confidentiality | 33 | How personal information about potential and enrolled participants will be collected, shared, and maintained in order to protect confidentiality before, during, and after the trial | Data management, sharing, and monitoring, page 25, Risk Management page 21 |
| Ancillary and post-trial care | 34 | Provisions, if any, for ancillary and post-trial care, and for compensation to those who suffer harm from trial participation  *Participants who experience injury in the course of study participation will be connected to appropriate clinical care. See IRB protocol uploaded with the submission.* |  |

*We strongly recommend reading this checklist in conjunction with the SPIRIT 2025 Explanation and Elaboration and the SPIRIT 2025 Expanded Checklist for important clarifications on all the items. We also recommend reading relevant SPIRIT extensions. See [www.consort-spirit.org](http://www.consort-spirit.org)

Citation: Chan A-W, Boutron I, Hopewell S, Moher D, Schulz KF, et al. SPIRIT 2025 statement: updated guideline for protocols of randomised trials. BMJ 2025;389:e081477. <https://dx.doi.org/10.1136/bmj-2024-081477>

© 2025 Chan A-W et al. This is an Open Access article distributed under the terms of the Creative Commons Attribution License (<https://creativecommons.org/licenses/by/4.0/>), which permits unrestricted use, distribution, and reproduction in any medium, provided the original work is properly cited.
